# Unlocking the secrets of peptide transport in wine yeast: insights into oligopeptide transporter functions and nitrogen source preferences

**DOI:** 10.1128/aem.01141-23

**Published:** 2023-10-16

**Authors:** Hidde Yaël Berg, Georg Arju, Carmen Becerra-Rodríguez, Virginie Galeote, Ildar Nisamedtinov

**Affiliations:** 1 Department of Chemistry and Biotechnology, School of Science, Tallinn University of Technology, Tallinn, Estonia; 2 Center of Food and Fermentation Technologies, Tallinn, Estonia; 3 Institute of Chemistry, University of Tartu, Tartu, Estonia; 4 SPO, Univ. Montpellier, INRAE, Institut Agro, Montpellier, France; 5 Lallemand, Inc., Montreal, Canada; The Pennsylvania State University, State College , Pennsylvania, USA

**Keywords:** oligopeptides, *Saccharomyces cerevisiae*, yeast physiology, oligopeptide transporters (opt), fungal oligopeptide transporters (fot), alcoholic fermentation

## Abstract

Oligopeptides from grape must represent a secondary source of nitrogen for yeasts to grow and carry out fermentation. *Saccharomyces cerevisiae* takes up oligopeptides from the environment through multiple oligopeptide transporters with different peptide length specificities. However, due to difficulties associated with the qualitative and quantitative measurement of peptides in natural matrices, peptide transporter specificities have been mostly researched in single peptide environments. Using a peptide mapping method, we monitored the relative consumption of peptides derived from a protein hydrolysate by a set of CRISPR-Cas9-engineered *S. cerevisiae* wine strains to study oligopeptide transporters from the Opt and Fot families. Results show that Opt2 can import peptides containing three to at least seven amino acid residues, which is a broader peptide length specificity than previously reported, while Opt1 activity was not sufficient to support growth on peptides as nitrogen source. Fot1, Fot2, and Fot3, previously referred to as di-tripeptide transporters in *S. cerevisiae* wine strains, could also import tetrapeptides. The consumption order of peptides was determined by the peptide length as higher chain length peptides were taken up by Opt2 only after most di-tetrapeptides were depleted from the media. Altogether, Fot and Opt2 activity assured completion of the fermentation process without necessarily requiring ammonia or free amino acids. Analysis of peptide transporter gene expression during fermentation showed an effect of SO_4_^2−^ not only on *OPT1* but also on *FOT* and supported the assumption of a possible interplay between Fot and Opt2 activities.

**IMPORTANCE:**

Limited nitrogen supply can prevent the completion of alcoholic fermentation. Supplementation through peptides as an alternative, natural source of nitrogen for yeast offers an interesting solution for this issue. In this work, the *S. cerevisiae* peptide transporters of the Opt and Fot families were studied. We demonstrated that Fot and Opt2 have a broader peptide length preference than previously reported, enabling yeasts to acquire sufficient nitrogen from peptides without requiring additional ammonia or amino acids to complete fermentation. On the contrary, Opt1 was unable to consume any peptide in the given conditions, whereas it has been described elsewhere as the main peptide transporter for peptides longer than three amino acid residues in experiments in laboratory conditions. This controversy signifies the need in applied sciences for approaching experimental conditions to those prevalent in the industry for its more accurate characterization. Altogether, this work provides further evidence of the importance of peptides as a nitrogen source for yeast and their consequent positive impact on fermentation kinetics.

## INTRODUCTION

Nitrogen is an essential element for yeast growth and activity during alcoholic fermentation. A deficiency of nitrogen in the environment inherently leads to a sluggish or incomplete fermentation (1). Additionally, the synthesis of a variety of volatile compounds, which shape the aroma profile of fermented beverages, is also affected by the availability and source of nitrogen (2, 3). In the fermentation industry and related applied research, the term yeast assimilable nitrogen (YAN) is typically used to refer to NH_4_^+^ and free amino acids (FAAs) which are metabolized by yeast, the content of which can vary to a great extent within different fermentation matrices (1). For example, the YAN values in natural grape musts remain in the range of 60 to 500 mg/L (4), depending on different factors such as grape variety, climate, or vinification conditions and practices. In addition to NH_4_^+^ and FAA, other sources of nitrogen, such as oligopeptides, can also be assimilated by yeast (5). The monitoring of nitrogen assimilation during fermentation using ^15^N-labeled NH_4_Cl and yeast hydrolysate showed that nitrogen derived from peptides constituted 40% of the yeast protein fraction (6). Furthermore, higher consumption of oligopeptides by yeasts during wine fermentation has demonstrated to positively impact cell viability, fermentation kinetics, and the production of volatile compounds (7 – 9). Thus, oligopeptides have an important anabolic role during fermentation. However, despite obvious advantages on fermentation kinetics, oligopeptide-derived YAN is rarely considered due to difficulties associated with their qualitative and quantitative analysis (10).

Oligopeptide import systems in yeast have been best characterized in *Saccharomyces cerevisiae*, the most widely used yeast species in industrial fermentations. These include seven individual transporters (Ptr2, Dal5, Opt1–2, and Fot1–3) which all mediate the uptake of oligopeptides across the plasma membrane in a proton-coupled mechanism. The first oligopeptide transporter identified in *S. cerevisiae* was the proton-dependent oligopeptide transporter Ptr2 [Pot/Ptr, transporter classification (TC) number: 2.A.17], which imports di- and tripeptides (11). The allantoate and ureidosuccinate permease Dal5 (TC 2.A.1.14.4) has also displayed dipeptide uptake activity (12). Fungal oligopeptide transporters (Fot1-Fot2 and Fot3) are found in several *S. cerevisiae* wine strains and other fungi, being experimentally characterized as di- and tripeptide importers as well (7, 13, 14); nonetheless, it has been speculated, albeit without solid evidence, that Fot may also transport longer-chain peptides (8, 15). To date, the uptake of longer-chain peptides has been shown to be mediated by the oligopeptide transporters Opt1 and Opt2 (TC 2.A.67). Although Opt1 orthologous transporters in the yeast species *Candida albicans* have been reported to import peptides of at least up to eight amino acid residues in length (16, 17), Opt1 and Opt2 in *S. cerevisiae* have been characterized as tetra- and pentapeptide transporters (18 – 21).

Regulation of oligopeptide transporters’ gene expression depends on the availability of assimilable nitrogen compounds in the medium. In yeast, the nitrogen catabolite repression (NCR) system involves several regulatory mechanisms for gene expression that repress the transcription of genes encoding for transporters of non-preferred nitrogen sources. A nitrogen source is considered to be preferred based on their capacity to support fast cell growth (e.g., NH_4_^+^, Glu, Gln, or Asn), while non-preferred sources trigger the de-repression of genes under control of the NCR (22 – 28). In *S. cerevisiae*, peptide transporter genes *PTR2*, *DAL5*, *OPT1*, and *OPT2* are all under the regulation of the NCR (11, 19, 26, 29 – 33). Interestingly, *OPT1* gene expression is specifically induced in sulfur starvation conditions, while the presence of sulfur-containing amino acids, Met and Cys, maintains the repression by NCR (24, 34). Regulation of *FOT* gene expression has not yet been thoroughly studied. Transcriptomic analyses on the *S. cerevisiae* wine strain EC1118, which contains *FOT1* and *FOT2*, revealed that these genes are upregulated during wine fermentation in nitrogen-limiting conditions (60–70 mg/L YAN), while being downregulated in nitrogen-rich synthetic musts (29, 30). A recent study on the expression levels of *FOT1*, *FOT2*, and *FOT3* in different *S. cerevisiae* wine strains during fermentation on natural and synthetic musts has shown that their expression levels are low overall, although they increase during the stationary phase in comparison to the mid-log phase (14). Furthermore, several binding motifs for the NCR regulators Gln3 or Cup9 were predicted in the promoter regions of *FOT* genes, corroborating the assumption that *FOT* gene expression is also regulated by the NCR (14).

Apart from amino acids, oligopeptides also exert a regulatory activity on the expression of peptide transporter genes. The uptake of dipeptides with basic and bulky amino acids at the N-terminal position (the “N-end rule dipeptides”) by Ptr2 upregulates its own expression by positive feedback (35). In this case, the N-end rule dipeptides bind to Ubr1, the mediator in ubiquitination processes, activating the degradation of Cup9, which otherwise acts as a transcriptional repressor of *PTR2* (36 – 39). Although Cup9 also represses the expression of *OPT2*, it is unknown whether the N-end rule peptides have the same inducing effect as in the case of *PTR2*. Contrary to the effect on *PTR2* and *OPT2*, Cup9 upregulates the expression of *DAL5*, while *OPT1* is not affected (12, 34). It is suggested that such divergent but complementary regulation of *PTR2*, *DAL5*, *OPT*, and *FOT* expression can allow yeasts to adapt to various environmental conditions and nutrient availabilities.

Substrate-dependent specificity of the different oligopeptide transporters has mostly been characterized by growth experiments with synthetic peptides as the sole nitrogen source. For example, tetra- and pentapeptide transport by Opt1 and Opt2 has been studied using peptide transporter gene(s) knockout (KO) strains cultured on different media containing single peptides, some of which exhibited a toxic/antimicrobial activity, such as KLAE (18, 21). In the case of toxic peptides, growth inhibition indicated the ability of the expressed peptide transporter to internalize the specific peptide. Another method used for studying transporter specificity of Dal5, Ptr2 (35), and Fot (13, 14) is the Biolog Phenotype MicroArrays platform (Hayward, CA, USA). While studies on single peptide consumption have contributed to our general understanding of oligopeptide transporter specificities, it has not provided information on the kinetics of peptide utilization during fermentation in complex media containing peptides of varying length and amino acid composition. We recently implemented a screening methodology for studying peptide assimilation by yeast in a synthetic medium supplemented with a single-protein hydrolysate with a characterized peptide composition (10). Using this approach, the relative consumption of over one hundred di- to hexapeptides were monitored during fermentations with three commercial *S. cerevisiae* wine strains. The results demonstrated different peptide uptake kinetics by these strains.

In the present work, the same methodology was applied to further elucidate the peptide-length specificity of Opt and Fot from *S. cerevisiae* during fermentation. For this purpose, we have worked with CRISPR-Cas9-engineered strains expressing different sets of oligopeptide transporters genes (14). The impact of peptide uptake by Opt and Fot on fermentation kinetics in media containing different concentrations and sources of nitrogen was then investigated. Finally, we analyzed *FOT* and *OPT* gene expression during fermentation, with a particular focus on the *OPT1* gene and its expression pattern in sulfur-limited media. This work deepens the characterization on Opt and Fot transporters in *S. cerevisiae*, providing new insights on the important role of peptides during fermentation under enological conditions.

## RESULTS

### Effect of the different nitrogen sources on cell growth and fermentation kinetics

To characterize the different peptide transporters and their effect on the yeast performance in a peptide rich environment, a set of CRISPR-Cas9-engineered knockout strains for single or multiple peptide transporter genes (Table 1) were used in fermentation experiments. Fermentations were conducted in three media containing 220 g/L of fermentable sugars consisting of glucose and fructose in equimolar concentrations but with different conditions of nitrogen supply (Materials and Methods; Table 2). Medium NA100 contained ~100 mg/L YAN from NH_4_^+^ and FAA, medium NAP200 had an additional ~100 mg/L nitrogen added from peptides, and medium P200 contained ~200 mg/L nitrogen delivered solely from peptides. The biomass density (OD_600_) and CO_2_ production were monitored during 12 days of fermentation (see Materials and Methods).

**TABLE 1:** Oligopeptide transporter knockout/knockin strains created from *Saccharomyces cerevisiae* strain 59A (haploid derivative of wine strain EC1118) used in this study^*a*^

Strain name	Genotype	Purpose	Reference/source
59A (wt)	MATa ; ho; AMN1::loxP	Wild-type strain; control	Marsit et al. (7)
PepKO	MATa ; ho; AMN1::loxP ; FOT1-2::loxP-kanMx-loxP ; dal5; opt1; opt2	Control, complete KO for oligopeptide transport	Becerra-Rodríguez et al. (14)
Opt1	MATa ; ho; AMN1::loxP ; FOT1-2::loxP-kanMx-loxP ; dal5 ;opt2	Functionality of Opt1	This study
Opt2	MATa ; ho; AMN1::loxP ; FOT1-2::loxP-kanMx-loxP ; dal5 ;opt1	Functionality of Opt2	This study
opt1Δ	MATa ; ho; AMN1::loxP; opt1	opt1 KO	This study
opt2Δ	MATa ; ho; AMN1::loxP; opt2	opt2 KO	This study
Fot1	MATa ; ho; AMN1::loxP ; FOT1-2::FOT1 ; dal5; opt1; opt2	Functionality of Fot1	Becerra-Rodríguez et al. (14)
Fot2	MATa ; ho; AMN1::loxP ; FOT1-2::FOT2 ; dal5; opt1; opt2	Functionality of Fot2	Becerra-Rodríguez et al. (14)
Fot3	MATa ; ho; AMN1::loxP ; FOT1-2::FOT3 ; dal5; opt1; opt2	Functionality of Fot3 (from *S. cerevisiae* strain K1)	Becerra-Rodríguez et al. (14)
Fot1Fot2	MATa ; ho; AMN1::loxP ; dal5 ; opt1 ; opt2	Functionality Fot1 and Fot2 together	Becerra-Rodríguez et al. (14)
fot1fot2Δ	MATa ; ho; AMN1::loxP ; FOT1-2::loxP-kanMx-loxP	fot KO/functionality of Opt1 and Opt2 together	Becerra-Rodríguez et al. (14)

^*a*^ Strains were selected to focus this study on Fot and Opt oligopeptide transporters.

**TABLE 2:** Nitrogen composition of the media used in the study^*a*^

	NA100: (NH_4_^+^ and AAs)	NAP200: (NH_4_^+^, AAs, and Peptides)	P200 and P200-SO_4_: (Peptides only)
NH_4_^+^	30	30	–
Amino acids	78	82	8^*b*^
Peptides	–	100	200
Total	108	212	208

^*a*^ The four media contained 220 g/L of glucose and fructose in equimolar concentrations. All values are displayed in milligram nitrogen per liter. NA100 and NAP200 contained another 17 mg/L nitrogen from proline; however, this amino acid was not taken into account as it is not assimilated by yeasts during fermentation (40).

^*b*^ Nitrogen deriving from free amino acids in the bovine serum albumin hydrolysate.

**Fig 1:**
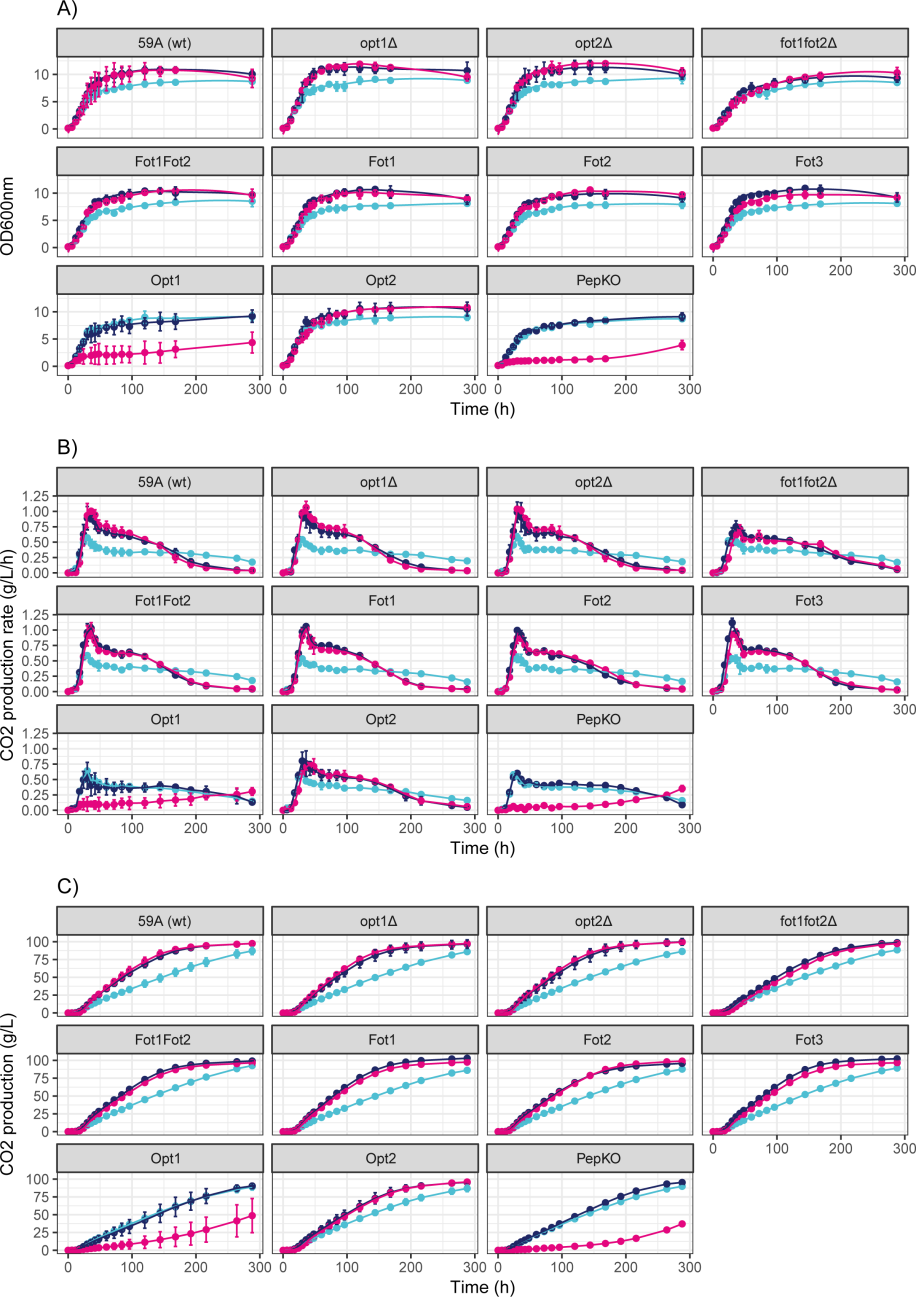
Growth and fermentation kinetics of the set of strains in different nitrogen conditions. Strain denominations can be found in Table 1. The NA100 condition is represented in light blue; NAP200 condition, in dark blue; and P200 condition, in pink. (**A**) Cell growth during fermentation. (**B**) Rate of CO_2_ production over time. (**C**) CO_2_ production over time.

**Fig 2:**
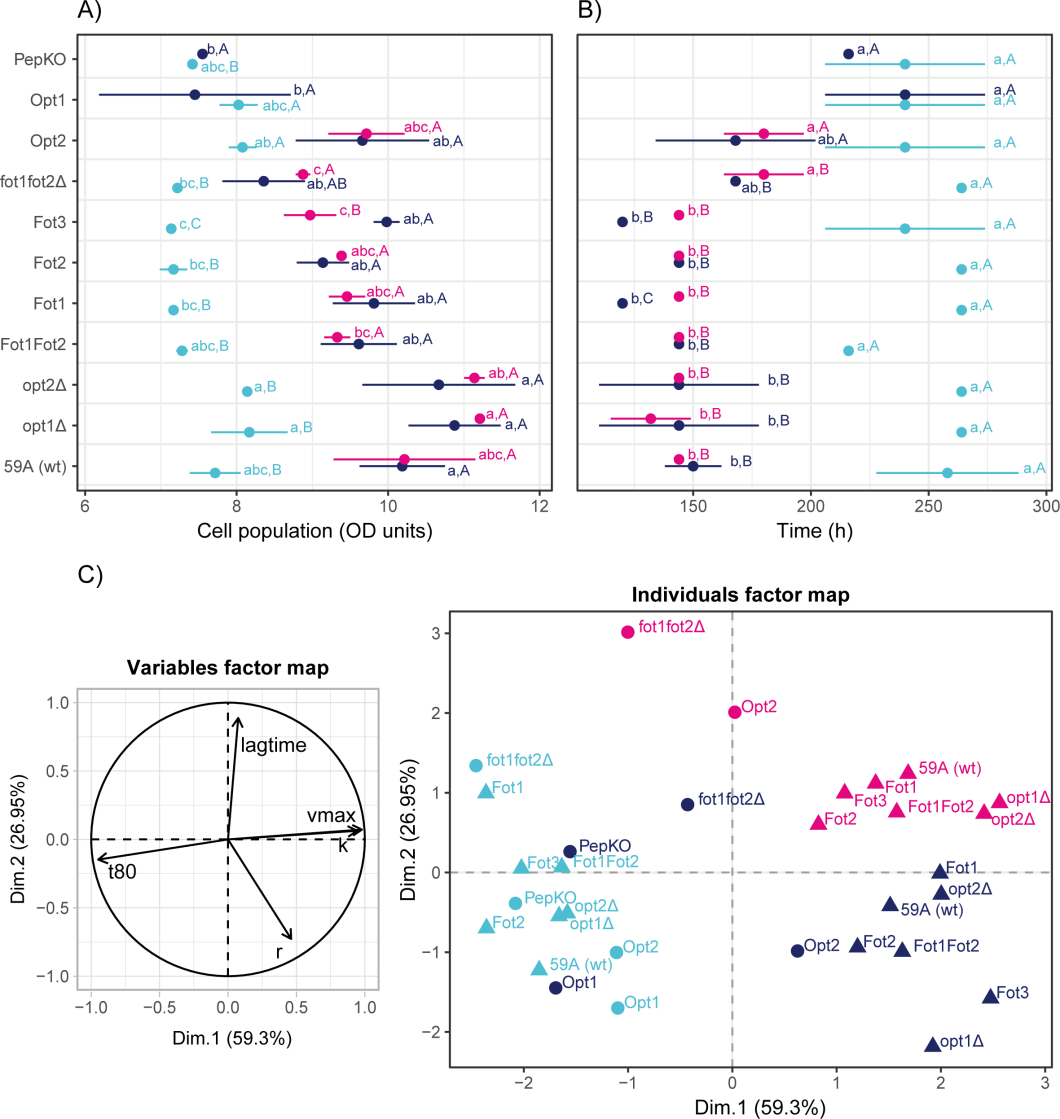
Growth and fermentation parameters with different nitrogen conditions. The NA100 condition is represented in light blue; NAP200 condition, in dark blue; and P200 condition, in pink. Letters in maximum cell population (**A**) and time to reach 80% of attenuation (**B**) denote the statistical groups from Tukey’s tests (*P* < 0.05), with lower-case letters indicating different groups of strains per medium and upper-case letters indicating the groups of media for every strain. (**C**) Principal component analysis of growth and fermentation parameters from the different strains in the three media. t80, time to reach 80% of attenuation; vmax, maximum CO_2_ production rate; lagtime, time to produce 1 g of CO_2_; k, maximum cell population; r, maximum growth rate. Triangles and circles in the individuals factor map respectively represent those strains that have or do not have Fot.

In fermentations with NH_4_^+^ and FAA as the sole nitrogen source (NA100), all strains displayed similar profiles of cell growth and fermentation kinetics (Fig. 1). In general, doubling the YAN concentration by the addition of peptides (NAP200 and P200) increased the maximum cell population reached by the strains when compared to the NA100 medium (Fig. 1A and 2A). The exceptions were the PepKO, Opt1, and, to a lesser extent, fot1fot2Δ and Opt2 strains. In the case of the fot1fot2Δ and Opt2 strain, an increase in the maximum cell population by the addition of peptides was observed; however, it was not significantly different from the value reached in NA100. By contrast, the growth of the PepKO and Opt1 strains was not affected by the doubling of YAN with peptides. Indeed, both strains could not grow in the P200 condition (Fig. 1), which suggests that the Opt1 strain, like the PepKO strain, is not able to utilize peptides as a nitrogen source. Notably, both strains showed an atypical increase in the biomass density and CO_2_ production rate at the endpoint of the experiment in the P200 medium (Fig. 1A and B) which may have been caused by the autolysis of part of the cell population and consequential release of assimilable FAA for growth. Due to this deviant behavior, the PepKO and Opt1 strains in the P200 condition were excluded from further statistical analysis of growth and fermentation parameters (Fig. 2).

The increase in the maximum cell population is negatively correlated with the time to reach 80% of attenuation, determined based on the maximum theoretical CO_2_ production from glucose and fructose (2 mol CO_2_/mol glucose/fructose) (Fig. 2B and C). This parameter was chosen as not all fermentations reached 100% of attenuation during the set experimental time. While all strains reached 80% of attenuation in the NA100 condition within ~250 hours on average, this point was reached within ~170 hours or less when the YAN content was doubled with the addition of peptides. Again, this tendency was more pronounced in those strains containing at least one *FOT*, which all reached 80% of attenuation before 150 hours in NAP200 and P200 media. By contrast, the PepKO and Opt1 strains reached 80% of attenuation in the NAP200 medium within similar time as the NA100 condition, which supports the assumption that these two strains are unable to utilize peptides. Similar results were obtained for the maximum rate of CO_2_ production (Vmax), as this parameter positively correlated with the maximum cell population and negatively correlated with the time to reach 80% of attenuation (Fig. 2C).

In addition to the maximum cell population, the time to reach 80% of attenuation and Vmax, the maximum growth rate, and the time to produce 1 g of CO_2_ (Table S1 through S5) were considered to further investigate the data set by a principal component analysis (Fig. 2C). Strains across different media clearly grouped according to the time to reach 80% of attenuation and Vmax, although there were some outliers. The PepKO and Opt1 strains in the NAP200 medium clustered with the NA100 group, supporting the assumption that the Opt1 transporter does not contribute to the uptake of peptides in the 59A strain. The fot1fot2Δ strain in the NAP200 medium did not cluster with the rest of the NAP200 group. The Opt2 strain in NAP200 was the only strain not containing *FOT* that clustered together with the *FOT*-containing strains in the same medium. The Opt2 and fot1fot2Δ strains were more distinguishable in the P200 medium. These results suggest that Opt2 is the only peptide transporter able to compensate with its activity for the lack of Fot in the 59A strain. Yet, the peptide import by Opt2 did not have the same impact on growth and fermentation kinetics as Fot.

### Uptake of oligopeptides by Opt and Fot transporters

**Fig 3:**
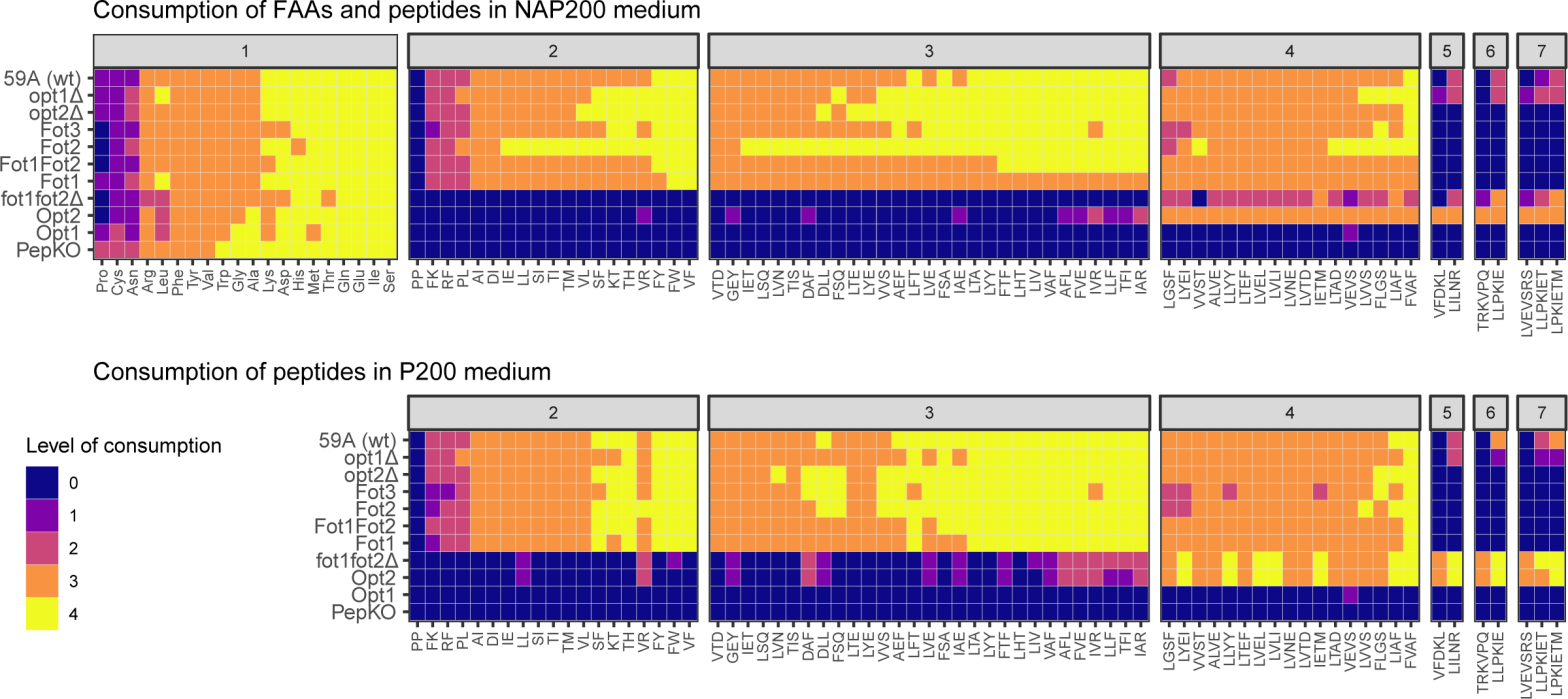
Consumption of FAA and peptides in NAP200 and P200 media. Peptides are grouped according to their number of amino acids residues. 1, FAAs; 2, dipeptides; 3, tripeptides; 4, tetrapeptides; 5, pentapeptides; 6, hexapeptides; 7, heptapeptides. The area under the curve (AUC) was calculated for the relative abundance curve of each peptide and FAA (Fig. S1 to S4). The AUC values were compared to a virtual negative control (100% abundance over 72 hours). The consumption of a particular peptide or FAA is level 0 when its abundance AUC is equal or higher than 80% of the negative control AUC; level 1, 60%–80%; level 2, 40%–60%; level 3, 20%–40%, and level 4 when the peptide abundance AUC is lower than 20% of the control AUC.

The consumption of peptides by the yeast strains in the NAP200 and P200 media was monitored during the first 72 hours of fermentation (Fig. 3; Fig. S1 to S6). In addition, the changes in the FAA concentration in the NAP200 medium were measured during the same time period (Fig. 3; Fig. S6). The consumption of FAA in the NAP200 medium was similar in most strains, indicating that the presence or absence of the different peptide transporters did not influence amino acid consumption. Only the PepKO strain showed higher consumption levels for FAA. This strain is incapable of peptide transport, and faster FAA consumption might have occurred to compensate for the lack of oligopeptide transport.

There was a clear distinction in the consumption of peptides based on their length. Those strains containing *FOT* (59A, opt1Δ, opt2Δ, Fot1Fot2, Fot1, Fot2, and Fot3) were all able to consume di-, tri-, and tetrapeptides (Fig. 3; Fig. S1 to S3; Fig. S5 and S6). Most di- to tetrapeptides were fully consumed between 18 and 36 hours of fermentation, even in presence of FAA and NH_4_^+^. The consumption of dipeptides with amino acid composition FK, RF, and PL by *FOT*-containing strains only started after depletion of the other dipeptides (at 18–24 hours) and were fully consumed after 72 hours in both media. Conversely, the strains containing *OPT2* and not *FOT* (fot1fot2Δ and Opt2) consumed tetra-, penta-, hexa-, and heptapeptides and only a few di- and tripeptides, with generally higher consumption levels in the P200 medium than in the NAP200. The wild-type strains 59A and opt1Δ, both containing *OPT2*, were also able to consume penta- to heptapeptides, although to a lesser extent than the fot1fot2Δ and Opt2 strains. As was expected from the fermentation kinetics analysis of the PepKO and Opt1 strains on NAP200 and P200 medium, neither of the strains were able to consume peptides. The only peptide that was not consumed by any of the strains in any condition was the dipeptide PP. These results re-define the current knowledge on the peptide length specificity of Fot, which goes up to tetrapeptides, and Opt2 in the wine strain 59A as the main transporter of peptides with a chain length containing four and more amino acids.

Strains expressing only Fot displayed similar profiles of di-, tri-, and tetrapeptide consumption in both the NAP200 and P200 conditions (Fig. 3; Fig. S5 and S6). However, the Fot2 expressing strain showed a more active consumption of di-, tri-, and tetrapeptides in the NAP200 condition than the other single-Fot strains, although the profile of consumption remained very similar (Fig. S5). This result indicates that the availability of FAA/NH_4_^+^ had limited influence on the specificity and activity of Fot.

Compared to Fot, the uptake of di- and tripeptides by the Opt2 transporter was limited. Out of the 19 dipeptides and 29 tripeptides of which the consumption was followed in this work, the Opt2 strain consumed only 1 or 2 dipeptides and 9 or 13 tripeptides in the NAP200 and P200 media, respectively (Fig. 3). Moreover, none of these di- and tripeptides were fully consumed during the first 72 hours of fermentation. By contrast, all tetra-, penta-, hexa-, and heptapeptides were fully consumed after 24–36 hours in both NAP200 and P200 media by the Opt2 strain. These results were corroborated by a similar peptide consumption profile observed with the fot1fot2Δ strain (containing *DAL5*, *OPT1*, and *OPT2*) when peptides were used as the sole nitrogen source (P200). However, in the NAP200 medium, the Opt2 and fot1fot2Δ strains showed a different uptake of peptides. First, di- and tripeptides were not consumed at all by the fot1fot2Δ strain (Fig. 2). Second, the consumption of tetra- to heptapeptides by the fot1fot2Δ strain only started after 24 hours when ~75% of FAA had been consumed (Fig. S3 and S4). This was different from the Opt2 strain, which took up these peptides much earlier. A similar activity of penta- to heptapeptide uptake by Opt2 as in the fot1fot2Δ strain was observed in the wild-type strain (59A) and opt1Δ strain. In all these strains, the uptake of penta- to heptapeptides by Opt2 was slower and started after depletion of most of the di-, tri-, or tetrapeptides. Thus, the uptake of longer peptides by Opt2 might be secondary to the uptake of FAA and smaller peptides in the presence of other peptide transporters.

### Fermentations under SO_4_^2^− limitation and expression of oligopeptide transporter genes

**Fig 4:**
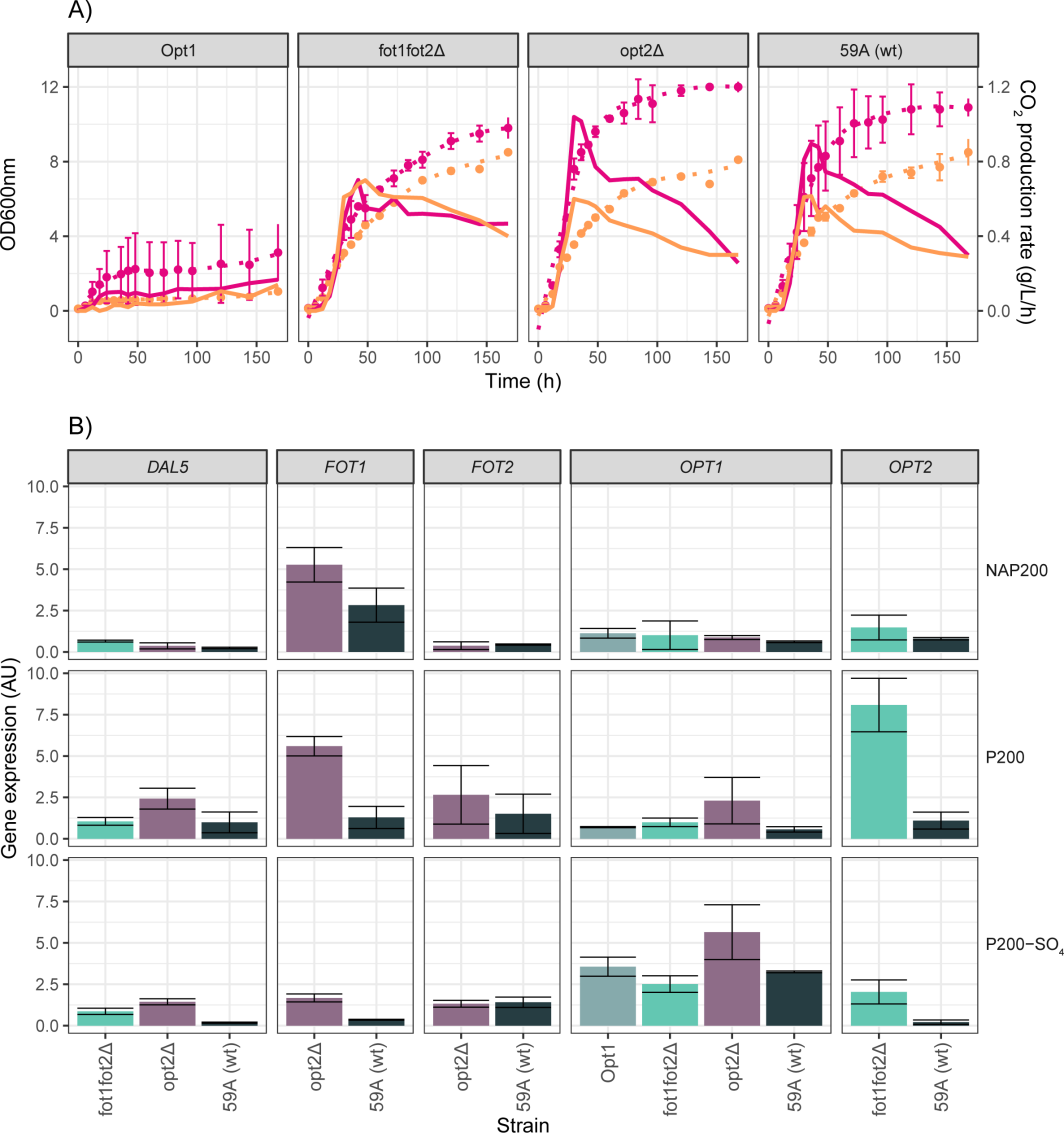
Influence of sulfur limitation on fermentation kinetics and the expression of peptide transporter genes. (**A**) Comparison of biomass production (OD_600_, dashed line) and CO_2_ production rate (straight line) between the strains grown in P200 (pink) or P200-SO_4_ (orange). (**B**) Expression of *DAL5*, *FOT1*, *FOT2*, *OPT1*, and *OPT2* in the *OPT1*-containing strains grown in the NAP200, P200, and P200-SO_4_ media, measured at 48 hours of fermentation.

A quantitative real-time polymerase chain reaction (RT-PCR) analysis was performed to study oligopeptide transporter gene expression during fermentation with different sources of nitrogen. To test the effect of the sulfur concentration on the expression of peptide transporter genes, as it was described for *OPT1* (34), fermentations were carried out on a version of the P200 medium with a reduction of 87% in SO_4_^2−^ salts (medium P200-SO4; Table 2). The strains selected for this experiment were those containing the *OPT1* gene (59A, opt2Δ, fot1fot2Δ, and Opt1 strains). Yeast growth and CO_2_ production rate in the P200-SO4 medium were monitored for 168 hours and compared to those in the P200 medium (Fig. 4A). Strains 59A and opt2Δ had lower CO_2_ production rates in the P200-SO4 medium in comparison to P200. This difference between the two media was also observed in growth, with lower biomass formation in the P200-SO4 medium by the end of the experiment. While the growth of the fot1fot2Δ strain in the P200-SO4 medium was also lower than in P200, the fermentation rate was not as affected as it was for the *FOT*-containing strains (59A and opt2Δ). No differences were detected between the P200 and P200-SO4 media for the Opt1 strain, due to the growth defect of this strain with peptides as the sole nitrogen source.

Gene expression was analyzed in NAP200, P200, and P200-SO4 at two different points of fermentation: 18 hours, which roughly corresponds to the beginning of the exponential growth phase, and 48 hours, which coincides with the beginning of the stationary phase (Fig. 4B; analysis of variance by Tukey’s tests can be found in Table S6). The expression of all peptide transporter genes was higher at 48 hours than at 18 hours of fermentation. This was the case for all the strains and media tested, with values of normalized expression at 18 hours not higher than 0.7 (AU). Therefore, differences in gene expression were further analyzed at 48 hours (Fig. 4B). As expected, the expression of *OPT1* was higher in the medium with a reduced concentration of SO_4_^2−^ in all the strains, which indicates that the gene is subjected to mechanisms of expression control as it has been previously reported (34). Moreover, the overexpression of *OPT1* was even more evident in the opt2Δ strain, suggesting that *OPT1* expression is under the control of *OPT2*. However, the overexpression of *OPT1* in the absence of *OPT2* was not observed in the Opt1 strain, probably due to the growth defect of this strain when peptides were the sole nitrogen source (Fig. 4A).

The *OPT1* gene in the 59A and Opt1 strains was sequenced to verify the possible gene variant in 59A and its peptide transporter KO derivatives. The *OPT1* gene sequence was identical in 59A and its derivative Opt1 strain (GeneBank accession number: OR468328). Likewise, the *OPT1* gene sequence in 59A and Opt1 strains was identical to the sequence in the genome of EC1118 (NCBI entry: FN393075.2, positions 35292 to 37691). The *OPT1* gene in 59A/EC1118 shares 99.25% of sequence identity with the reference strain S288C (NM_001181645.1) (Fig. S7), which translates to 100% identity at the protein sequence level. Therefore, this result confirmed that the *OPT1* gene in 59A/EC1118 does not contain any mutation that could turn the protein non-functional.

The *OPT2* gene was more highly expressed in the P200 medium in comparison to NAP200 or P200-SO4, both in the 59A and fot1fot2Δ strains. As peptides were the only source of nitrogen in P200, it seems that *OPT2* in this strain was repressed by the presence of NH_4_^+^ and FAA. This corroborates with lower peptide consumption levels by the fot1fot2Δ strain in NAP200 (Fig. 3). *OPT2* expression was also affected by the absence of sufficient SO_4_^2−^. Interestingly, the expression level of *OPT2* in the fot1fot2Δ strain was generally higher compared to that in the wild-type strain 59A. As Opt2 was the only active peptide transporter in this strain, higher expression of the gene might compensate for the lack of *FOT*. A similar trend was observed for *FOT1*, which was more expressed in the opt2Δ strain than in 59A. Interestingly, *FOT1* was significantly lower expressed in the P200-SO_4_, which indicates that *FOT1* expression may be affected by the concentration of SO_4_^2−^. In contrast, *FOT2* did not seem to respond to the absence of *OPT2* or the low SO_4_^2−^ concentration in the same way as *FOT1*. Similar to *OPT2*, higher expression levels of *FOT2* were observed in the absence of NH_4_^+^ and FAA (P200).

Despite the experimental setup not being focused on the dipeptide transporter *DAL5*, its expression was monitored along with *FOT* and *OPT* genes. The expression of *DAL5* was higher when peptides were the only source of nitrogen and for strains where either *OPT2* or *FOT* was missing.

## DISCUSSION

In this work, the role of yeast oligopeptide transporters of the Opt and Fot families in the assimilation of peptides during fermentation under different nitrogen conditions were studied. For this purpose, we worked with engineered strains derived from 59A, a haploid version of the commercial *S. cerevisiae* wine strain EC1118, which contains functional genes for Opt1, Opt2, Dal5, Fot1, and Fot2 oligopeptide transporters. The strains were engineered to either express or lack the expression of single peptide transporter genes (14). 59A and most of its derivative strains were able to complete fermentation with peptides as the sole nitrogen source, demonstrating that peptides can support yeast growth during fermentation without requiring NH_4_^+^ nor FAA. These results reinforce the assumption that peptides can represent a powerful resource to cope with problems during fermentations associated with nitrogen deficiency.

Nonetheless, not all the 59A-derivative strains could complete fermentation with peptides as the sole nitrogen source. These include the complete peptide transporter knockout strain (PepKO) and the strain with Opt1 as the sole peptide transporter. Opt1 was originally characterized as the tetra- and pentapeptide transporter in descendants of the reference strain *S. cerevisiae* S288C (18, 21). In those strains, the given transporter was overexpressed within a plasmid (18, 21). Opt1 is also a glutathione transporter, and it has been hypothesized that this function might be primary to peptide transport (20, 34). A potential incapacitating mutation of *OPT1* in 59A was excluded by Sanger sequencing, obtaining a translated protein sequence identical to the Opt1 sequence in S288C. In this study, the Opt1 strain did not consume any peptides, and the experiments did not include glutathione. Thus, the role of Opt1 in 59A and consequently EC1118 remains unclear. Nonetheless, the fot1fot2Δ strain has previously shown a growth defect in fermentations with glutathione as the sole nitrogen source in comparison with 59A (7), which in that work served as a validation of Fot as glutathione transporters but now indicates that the uptake of glutathione and other peptides by Opt1 may have a more regulatory role, e.g. by responding to oxidative stress or other stress-related events in the cell, rather than nutrients import.

Although the main factor contributing to the improvement of growth and fermentation kinetics was the increase in the nitrogen concentration, regardless of the type of nitrogen source, those strains containing *FOT* responded better when peptides were added to the medium. Fot, therefore, seem to be the most important peptide transporters in 59A, which supports previous characterization of these transporters in synthetic conditions, as well as with natural peptides from grape must (7, 14). Peptide analysis at different points of fermentation showed that 59A was able to consume a wide variety of peptides, ranging from di- to heptapeptides. Fot1, Fot2, and Fot3, previously characterized as di-tripeptide transporters (7, 13, 14), were also able to import tetrapeptides, while they did not transport penta-, hexa- or heptapeptides. Kinetics of peptide consumption during fermentation suggests that Fot are effective di-tetrapeptide transporters already active at the early stages of fermentation, even when preferable nitrogen sources (NH_4_^+^ and FAA) are also present. The strains expressing a single *FOT* also had different levels of peptide consumption in our experimental conditions, corroborating the results of Becerra-Rodríguez et al. (14). However, in contrast to the previously mentioned work, here, the Fot2 strain had the most active consumption of peptides instead of the strain containing both *FOT1* and *FOT2*. Discrepancies between the previous work and the present study could be due to the distinct types of peptides utilized. While Becerra-Rodríguez et al. monitored the consumption of single peptides through a colorimetric detection of yeast growth in laboratory conditions, this study carried out a semi-quantitative and qualitative analysis of the consumption of a complex mix of peptides derived from a bovine serum albumin (BSA) hydrolysate during model alcoholic fermentation under enological conditions. Therefore, the comparison of substrate specificity between the two studies is not pertinent.

As for Opt2, the strains expressing this gene while lacking *FOT* were also able to grow and carry out fermentation with peptides as a nitrogen source, although not to the same extent as *FOT*-containing strains. The distribution of nitrogen over the different peptide length groups is unknown, as only the relative decrease in abundance of the identified peptides could be followed. Therefore, it is not possible to state if the better response to the addition of peptides by the *FOT*-containing strains was caused by a higher efficiency of Fot or by a higher nitrogen content in the Fot-consumed peptide-length groups. We also determined a broader length specificity for Opt2, so far defined as a tetrapeptide transporter in *S. cerevisiae* (19). The strain with Opt2 as the sole peptide transporter gave preference to consuming tetra- to heptapeptides, while only partially consuming a few tripeptides and dipeptides. Indeed, the consumption of tetra- to heptapeptides by the Opt2 strain occurred within the first 36 hours of fermentation, regardless of the presence or absence of NH_4_^+^ and FAA. In contrast, the fot1fot2Δ strain, whose sole functional peptide transporter was proven to be Opt2, consumed tetra- to heptapeptides slower in the NAP200 medium compared to the P200. This result suggests that contrary to Fot, Opt2 oligopeptide transport activity may be affected by the presence of NH_4_^+^ and FAA when other peptide transporter genes are also present. Fot-mediated peptide transport occurred simultaneously, albeit with a slight delay, with FAA consumption. Most FAA and di- to tetrapeptides were completely consumed during the first 18–36 hours of fermentation by all strains containing *FOT*. By contrast, Opt2-associated peptide transport in both the 59A and opt1Δ strains occurred after most FAA and di- to tetrapeptides were consumed, which suggests a preference for the consumption of di-tetrapeptides by Fot over the uptake of longer peptides by Opt2. We hypothesize that there is a balance between Fot and Opt2 activity, where Fot-mediated di-tetrapeptide consumption is the main system for oligopeptide acquisition during the first stages of fermentation. Due to the broad peptide length specificity of Fot, yeast can take up a wide range of nitrogen sources for fast growth, which is needed to become the dominant organism in a competitive environment such as grape must. We suggest that Opt2 activity is then used to obtain additional nitrogen from larger peptides when more easily assimilable nitrogen sources are depleted.

The peptide mapping strategy applied in this study allows for reliable peptide identification at the level of peptide length and amino acid composition but not the sequence (10). On this level, peptides that were not taken up or taken up poorly by either Fot or Opt2 shared similar chemical properties with those that were fast consumed and could, therefore, not be differentiated. A more in-depth analysis of sequence-based uptake preferences would still require experiments with synthetic peptides in this case. Another possibility to infer peptide sequences would consist in using a protease of known cutting site specificity to digest BSA, such as trypsin or chymotrypsin. Peptide mass data can then be used for peptide fingerprint identification through *in silico* approaches.

The expression of peptide transporter genes during fermentation was influenced by the available nitrogen source(s), potentially via the NCR system. Notably, expression levels of *FOT2* in the 59A and opt2Δ strains were higher in the medium containing only peptides (P200) than in the medium which contained both peptides and FAA/NH_4_^+^. Similarly, *OPT2* in the fot1fot2Δ strain also had a lower expression level in the presence of NH_4_^+^ and FAA. This corresponded with the earlier-mentioned lower consumption levels of peptides by the fot1fot2Δ strain in the presence of NH_4_^+^ and FAA. This effect was absent in the Opt2 strain, where Opt2 was the sole peptide transporter, which raises the question whether either *OPT1* or *DAL5* was involved in the regulation of *OPT2*. For example, such regulation has been shown on *OPT1* by *OPT2* (34, 38). Wiles et al. (34) speculated that in the absence of one of the *OPT*, yeast might up-regulate the other *OPT* to compensate for weaker oligopeptide uptake potential (34). However, the lack of peptide consumption by the Opt1 strain does not support this hypothesis. Furthermore, *OPT2* was more highly expressed in the absence of *FOT1* and vice versa, which is consistent with our hypothesis of an interplay between Fot1 and Opt2 functionality.

When the concentration of sulfur, known to repress *OPT1* (34), was reduced by 87%, a more pronounced expression of *OPT1* could be observed in all strains. However, this did not lead to improved growth of the Opt1 strain in the P200-SO4 medium, while other strains were still able to grow. The fact that *OPT1* gene expression responded positively to sulfur limitation indicates that Opt1 inability to support growth as peptide transporter is not due to alterations associated with gene expression.

Interestingly, *FOT*-containing strains (opt2Δ and 59A) displayed lower fermentation rates and reached a lower biomass formation under sulfur-limiting conditions. Sulfur and nitrogen metabolism are linked, since sulfate assimilation is required for the biosynthesis of the sulfur-containing amino acids methionine and cysteine (41). Marsit et al. (9) observed an up-regulation of the genes involved in the synthesis of cysteine and methionine in the strain 59A when compared to fot1fot2Δ (9). In this work, they concluded that peptides consumed by Fot were incorporated into the glutamate node, increasing *de novo* amino acid and glutathione biosynthesis. Based on these results, we hypothesize that peptide consumption by Fot induces the up-regulation of genes involved in the synthesis of sulfur amino acids, but due to the lack of SO_4_^2−^ in the medium, there might be a metabolic imbalance that leads the cell to not grow or carry out fermentation properly. A deeper investigation into the link between peptide and sulfur metabolism is required to fully understand these mechanisms but was not within the scope of the current work.

This work has highlighted the importance of peptides as a nitrogen source for *S. cerevisiae* during fermentation. We demonstrated a broader peptide length specificity of Fot and Opt2 than previously reported. The results also showed that Opt2, not Opt1, was the main tetra- to heptapeptide transporter in the *S. cerevisiae* wine strain 59A. Furthermore, the complementary peptide uptake specificities of Fot and Opt2 and expression of the two at different phases of fermentation allowed yeast to consume preferred nitrogen sources in an orderly fashion. However, the fact that Opt1 as single available peptide transporter could not support growth, together with the inactivity of Ptr2 in this strain due to a gene truncation is indicative of the variability of peptide transporter functionality in yeast. Studies including different strains of *S. cerevisiae* are required to comprise the high intraspecific variability of *S. cerevisiae* and build a more global picture of peptide transport within the species.

## MATERIALS AND METHODS

### Strains

The oligopeptide transporter knockout strains derived from the *S. cerevisiae* strain 59A (haploid variant of the commercial wine strain EC1118) were produced in an earlier study (14). Since the purpose of that previous work was to characterize Fot, a strain expressing the *FOT3* gene from another wine strain was also included (Table 1).

The fot1fot2Δ strain was created by replacing the tandem genes *FOT1–FOT2* by a *KANMX4* cassette. Genes *OPT1*, *OPT2*, and *DAL5* were then deleted sequentially from the fot1fot2Δ strain using the CRISPR/Cas9 system. *PTR2* is not functional in 59A, and it was, therefore, not required to delete this gene (13). Deletion of *OPT1*, *OPT2*, and *DAL5* oligopeptide transporter genes in fot1fot2Δ resulted in the strain *opt1Δopt2Δdal5Δfot1fot2Δ::KANMX4*, which is a full knockout strain for oligopeptide transport (PepKO). Using CRISPR/Cas9, each *FOT* gene (*FOT1* and *FOT2* from EC1118 and *FOT3* from *S. cerevisiae* strain K1) was knocked-in as a substitution for the *KANMX4* cassette in the PepKO strain. In this way, all *FOT* genes were individually located in the original *FOT1–FOT2* locus and, therefore, were under the regulation of the *FOT2* promoter and *FOT1* terminator.

### Synthetic grape must

Fermentation experiments were carried out on synthetic grape must (SGM) (42). The initial SGM (NA100, Table 2) contained 220 g/L of glucose and fructose in equimolar concentrations and 108 mg/L of YAN from NH_4_^+^ and amino acids [except proline, which is not assimilated by yeasts during fermentation (40)]. The amount of nitrogen in the NA100 medium was deliberately low for the given concentration of fermentable sugars, since it subsequently served as a basis for the addition of 100 mg/L of nitrogen in the form of peptides from the BSA hydrolysate, constituting the NAP200 medium. The nitrogen source in the P200 medium contained only peptides from the BSA hydrolysate, added to reach c.a. 200 mg/L of nitrogen. To study the role of sulfur in the expression of *OPT1*, the P200-SO_4_ medium was prepared with the same peptide composition as in P200 but with 87% reduced concentration of sulfur (from K_2_SO_4_ and MgSO_4_). To compensate for the lack of potassium and magnesium in the P200-SO_4_ medium, KCl and MgCl_2_ were added to match the concentrations of these elements in NA100, NAP200, and P200. All media were filter sterilized using a 0.22-µm Steritop Vacuum Driven Disposable Filtration System (Merck-Millipore, Burlington, MA, USA) prior to yeast inoculation.

### Preparation of BSA hydrolysate

A BSA enzymatic hydrolysate was used as a source of peptides in the fermentation media. The hydrolysate was prepared according to a previously reported method (10). In addition to ultrafiltration of the hydrolysate using a Vivaflow 200 10,000 MWCO Hydrosart crossflow cassette (Sartorius, Göttingen, Germany), the hydrolysate was subsequently filtered using a vivaflow 200 2,000 MWCO Hydrosart crossflow cassette (Sartorius, Göttingen, Germany) to further concentrate the smaller MW peptide fraction (<2 kDa), potentially assimilable by yeast. The permeate fraction of the hydrolysate was then freeze dried and stored at −20°C until further use.

### Fermentation experiments

Each yeast inoculum was prepared from a single colony that was pitched to a shake flask containing yeast peptone dextrose medium (1% yeast extract, 2% bacteriological peptone, and 2% glucose). The shake flasks were incubated overnight at 30°C and 150 rpm. Cells were washed twice with equal volumes of sterile 0.9% NaCl prior to inoculation into fermenters to deliver 5 × 10^6^ cells/mL in a final volume of 100 mL of SGM. Fermentations were performed in duplicate at 24°C in 100-mL Pyrex bottles equipped with a GL45 open top PBT screw cap and PYREX Media Bottle Septum (Corning, Inc., Corning, NY, USA). A gas outlet was installed to prevent overpressure by piercing the septum with a Sterican Ø 0.8 × 40 mm single-use hypodermic needle (B. Braun, Melsungen, Germany) attached to a Millex-FG 0.2-µm hydrophobic PTFE filter (Merck KGaA, Darmstadt, Germany).

Samples (2 mL) were collected every 6 hours for the first 48 hours, then every 12 hours until 96 hours and finally every 24 hours until 168 hours. Monitoring of the CO_2_ production continued every 24 hours until the end point (288 hours). Two additional OD_600_ measurements were performed at 240 and 288 hours, and a final sample was taken at 288 hours. The biomass density of the samples was assessed by measuring the optical density at 600 nm using an Ultrospec 10 Cell Density Meter (Biochrom Ltd., Cambridge, UK). The specific production rate of CO_2_, monitored gravimetrically, was used as the main indicator of fermentation progress. After centrifugation at 9600 × *g* for 10 min at 4°C in a MicroCL 21R Microcentrifuge (Thermo Fisher Scientific, MA, USA), the supernatant and biomass pellet were separately stored at respectively −20°C and −80°C, until further analysis.

### Amino acid analysis

The amounts of free amino acids were analyzed on a Waters ACQUITY UPLC system (Waters Corporation, Milford, MA, USA) that was coupled to a TUV detector after derivatization using Waters AccQ-Tag chemistry (43).

### Peptide analysis

#### Sample preparation

For peptides analysis, the fermentation samples were first mixed (1:1) with methanol in BRANDplates pureGrade 96-Well Microplates (BRAND GMBH + CO KG, Wertheim, Germany). The plates were then centrifuged at 560 × *g* in a BioSan LMC-3000 centrifuge (Biosan) to remove the precipitate. Then, 20 µL from each well was transferred to a Waters Round well Polypropylene 350-µL 96-well Sample Collection Plate (Waters Corporation, Milford MA, USA) and diluted with MilliQ water (160 µL) to a final volume of 180 µL. All samples were spiked with 0.5 ppm caffeine (20 µL) to a final volume of 200 µL. Caffeine was used as an internal standard (reference housekeeping ion) during subsequent analysis.

#### Liquid chromatography mass spectrometry (ultra-high-pressure liquid chromatography ion mobility separation-enabled high-resolution mass spectrometry [UHPLC-IMS-HRMS])

Peptides were analyzed using the methodology described in reference (10). Briefly, Waters I-Class Plus (SM-FL) UPLC system (Waters Corporation, Milford, MA, USA) was used coupled with a Waters Vion IMS-QTof Mass Spectrometer equipped with a LockSpray II Exact Mass source enclosure and MKII tool-free ESI probe assembly directly connected to the column outlet. Nitrogen was used as collision gas. The instrument was controlled by Waters UNIFI 1.9.4 (3.1.0, Waters Corporation, Milford, MA, USA).

The instrument was operated in positive polarity, sensitivity mode (32,000 FWHM at 556.2766 *m/z*), and labile ion mobility tune. The analysis type was set as Peptide Map (IMS), and the experiment type was set to MSe. Data were acquired in HDMSe mode with a scan time of 0.165 s. The following manual quadrupole profile was used: mass 150/250/450 (*m/z*), dwell time 60/20 (% scan time), ramp time 10/10 (% scan time).

The injection volume was 5 µL. Full loop (5 µL) injection mode was used with 3 vol overfill. Samples were analyzed using an Acquity UPLC HSS T3 Column (1.8 µm, 1 × 150 mm, Waters Corporation, Milford, MA, USA) kept at 45°C. The initial flow rate was 0.2 mL/min. The gradient was as follows: a 0–0.5 min hold at 3% B; 0.5–5.5 min linear gradient, 3%–28% B; 5.5–7 min linear gradient, 28%–95% B; 7–7.5 min hold at 95% B accompanied by a linear flow rate increase of 0.2–0.3 mL/min; 7.5–7.6 min linear gradient, 99%–3% B accompanied by a linear flow rate decrease of 0.3–0.2 mL/min; and 7.6–10 min hold at 3% B.

#### Data processing of amino acid and peptide data

The peptide mapping for peptide identification in the purified BSA hydrolysate fraction was conducted according to the method used by reference (10). Consumption of peptides up to seven amino acids in length was studied. A reduction from the initial peptide signal response of at least 20% was regarded as consumption, while peptides with an increase of >20% from the initial peptide signal response were excluded. A list with the mass spectrometric data of the final selection of di-heptapeptides included in this study can be found in Table S7. All subsequent peptide signal responses exceeding the response of the peptide at the starting point were normalized to an abundance of 100% with respective relative standard deviations. Relative consumption curves for FAAs were also calculated based on their abundance at the start of fermentation. The area under the curve (AUC) was calculated for the relative abundance curve of each peptide and FAA (Fig. S1 through S4). The AUC values were compared to a virtual negative control (100% abundance over 72 hours). The consumption of a particular peptide or FAA is in level 0 when its abundance AUC is equal or higher than 80% of the negative control AUC; level 1, 60%–80%; level 2, 40%–60%; level 3, 20%–40%; and level 4 when the peptide abundance AUC is lower than 20% of the control AUC.

### Gene expression analysis

Expression levels of peptide transporter genes *DAL5*, *OPT1*, *OPT2*, *FOT1*, and *FOT2* were analyzed in technical duplicates in the 18- and 48-hour samples from fermentation experiments in NAP200, P200, and P200-SO4. RNA was extracted using a phenol-chloroform method. For this, RNA buffer (120 µL, 50 mM TRIS-HCL, pH 7.4; 100 mM NaCl; 10 mM EDTA) and glass beads were added to frozen biomass samples. Cells were then disrupted in a bead mill Disruptor Genie (Scientific Industries, Inc., Bohemia, NY, USA) for 3 minutes with 1-minute intervals for cooling on ice. RNA buffer + 1.3% SDS (450 µL) and acid phenol (450 µL, pH 5) were subsequently added to the samples, followed by another cell disruption cycle. Samples were then centrifuged for 10 min at 21,100 × *g*. The upper phase was transferred to a fresh Eppendorf tube. Acid phenol (300 µL) and chloroform (300 µL) were added, after which the samples were vortexed and centrifuged for 10 minutes at 21,100 × *g*. The upper layer (500 µL) was then transferred to a new 1.5-mL Eppendorf tube. RNA was allowed to precipitate by adding 4 M NaCl (20 µL) and 96% ethanol (1 mL) and subsequently letting it stand for 30 min at −20°C. A pellet of RNA was obtained after centrifugation at 21,100 × *g* for 10 minutes and subsequent washing with 70% ethanol (150 µL). The RNA pellet was then resuspended in RNase/DNase-free water (30–50 μL) to obtain the final RNA extract. Total RNA was treated with a gDNA Removal Kit (Jena Bioscience, Jena, Germany) and quantified using the Invitrogen Qubit RNA Broad Range (BR) Assay Kit (Thermo Fisher Scientific, Waltham, MA, USA). cDNA was obtained by RT-PCR from 1 µg of RNA using the FIREScript RT cDNA synthesis KIT (Solis BioDyne, Tartu, Estonia). For RT-PCR, 5× HOT FIREPol EvaGreen qPCR Mix Plus (Solis BioDyne, Tartu, Estonia) was used. RT-PCR was conducted on a qTOWER G^3^ (Analytik Jena AG, Jena, Germany) system. A list of the primers used can be found in Table 3. Gene expression values were normalized to those of the housekeeping gene *ACT1*.

**TABLE 3:** Real-time polymerase chain reaction (RT-PCR) primers used in this study

Primer name	Sequence
ACT1-FW	CCACCATGTTCCCAGGTATT
ACT1-RV	CCAATCCAGACGGAGTACTT
DAL5-FW	ATCACCCAACGGTAAAATTG
DAL5-RV	ATCTTCTTCTGGTGTCACTT
OPT1-FW	CCACCAAGCACACCTTATAAC
OPT1-RV	ATTGCCACACCTGCTTCAACA
OPT2-FW	CCTGATGCTGTGACCTACTAT
OPT2-RV	ATGCATGCGCCTATCAACCAA
FOT1-FW	GCGGTTGGTTGTTGAACTTT
FOT1-RV	GGGCAGTGCTCAGAAGAATC
FOT2-FW	CGAGGGCTTATGACGAGGTA
FOT2-RV	GATCCCAGCGTAGTGGACAT

### Data treatment and statistical analysis

Data were treated and analyzed using R v4.2.1 (R Core Team 2022) and RStudio (RStudio Team 2022). The growth rate and maximum cell population were estimated by fitting the optical density curves with the summarizegrowth R function from the Growthcurver package (44). Significance of the analysis of variance between mean values was assessed using Tukey HSD method.

## Data Availability

Data files and the R script for data treatment and analysis can be found at https://zenodo.org/record/8278654.

## SUPPLEMENTAL MATERIAL

Supplemental Tables and Figures aem.01141-23-s0001.docx Tables S1-7 and Figures S1-7.

ASM does not own the copyrights to Supplemental Material that may be linked to, or accessed through, an article. The authors have granted ASM a non-exclusive, world-wide license to publish the Supplemental Material files. Please contact the corresponding author directly for reuse.

## References

[1] Bell S-J , Henschke PA . 2005. Implications of nitrogen nutrition for grapes, fermentation and wine. Aust J Grape Wine Res 11:242–295. doi:10.1111/j.1755-0238.2005.tb00028.x

[2] Hazelwood LA , Daran J-M , van Maris AJA , Pronk JT , Dickinson JR . 2008. The ehrlich pathway for fusel alcohol production: a century of research on Saccharomyces cerevisiae metabolism. Appl Environ Microbiol 74:2259–2266. doi:10.1128/AEM.02625-07 18281432 PMC2293160

[3] Thompson-Witrick KA , Pitts E . 2020. Nitrogen content in craft malts: effects on total ester concentration in beer. J Am Soc Brew Chem 78:308–313. doi:10.1080/03610470.2020.1778432

[4] Bely M , Sablayrolles J-M , Barre P . 1990. Automatic detection of assimilable nitrogen deficiencies during alcoholic fermentation in oenological conditions. J Ferment Bioeng 70:246–252. doi:10.1016/0922-338X(90)90057-4

[5] Mo F , Zhao H , Lei H , Zhao M . 2013. Effects of nitrogen composition on fermentation performance of brewer's yeast and the absorption of peptides with different molecular weights. Appl Biochem Biotechnol 171:1339–1350. doi:10.1007/s12010-013-0434-5 23955296

[6] Kevvai K , Kütt M-L , Nisamedtinov I , Paalme T . 2016. Simultaneous utilization of ammonia, free amino acids and peptides during fermentative growth of Saccharomyces cerevisiae. J Inst Brew 122:110–115. doi:10.1002/jib.298

[7] Marsit S , Mena A , Bigey F , Sauvage F-X , Couloux A , Guy J , Legras J-L , Barrio E , Dequin S , Galeote V . 2015. Evolutionary advantage conferred by an eukaryote-to-eukaryote gene transfer event in wine yeasts. Mol Biol Evol 32:1695–1707. doi:10.1093/molbev/msv057 25750179 PMC4476156

[8] Duc C , Maçna F , Sanchez I , Galeote V , Delpech S , Silvano A , Mouret J-R . 2020. Large-scale screening of thiol and fermentative aroma production during wine alcoholic fermentation: exploring the effects of assimilable nitrogen and peptides. Fermentation 6:98. doi:10.3390/fermentation6040098

[9] Marsit S , Sanchez I , Galeote V , Dequin S . 2016. Horizontally acquired oligopeptide transporters favour adaptation of Saccharomyces cerevisiae wine yeast to oenological environment. Environ Microbiol 18:1148–1161. doi:10.1111/1462-2920.13117 26549518

[10] Arju G , Berg HY , Lints T , Nisamedtinov I . 2022. Methodology for analysis of peptide consumption by yeast during fermentation of enzymatic protein hydrolysate supplemented synthetic medium using UPLC-IMS-HRMS. Fermentation 8:145. doi:10.3390/fermentation8040145

[11] Perry JR , Basrai MA , Steiner HY , Naider F , Becker JM . 1994. Isolation and characterization of a Saccharomyces cerevisiae peptide transport gene. Mol Cell Biol 14:104–115. doi:10.1128/mcb.14.1.104-115.1994 8264579 PMC358361

[12] Cai H , Hauser M , Naider F , Becker JM . 2007. Differential regulation and substrate preferences in two peptide transporters of Saccharomyces cerevisiae. Eukaryot Cell 6:1805–1813. doi:10.1128/EC.00257-06 17693598 PMC2043388

[13] Damon C , Vallon L , Zimmermann S , Haider MZ , Galeote V , Dequin S , Luis P , Fraissinet-Tachet L , Marmeisse R . 2011. A novel fungal family of oligopeptide transporters identified by functional metatranscriptomics of soil eukaryotes. ISME J 5:1871–1880. doi:10.1038/ismej.2011.67 21654847 PMC3223307

[14] Becerra-Rodríguez C , Taghouti G , Portier P , Dequin S , Casal M , Paiva S , Galeote V . 2021. Yeast plasma membrane fungal oligopeptide transporters display distinct substrate preferences despite their high sequence identity. J Fungi (Basel) 7:963. doi:10.3390/jof7110963 34829250 PMC8625066

[15] Becerra-Rodríguez C , Marsit S , Galeote V . 2020. Diversity of oligopeptide transport in yeast and its impact on adaptation to winemaking conditions. Front Genet 11:602. doi:10.3389/fgene.2020.00602 32587604 PMC7298112

[16] Reuss O , Morschhäuser J . 2006. A family of oligopeptide transporters is required for growth of candida albicans on proteins. Mol Microbiol 60:795–812. doi:10.1111/j.1365-2958.2006.05136.x 16629678

[17] Dunkel N , Hertlein T , Franz R , Reuß O , Sasse C , Schäfer T , Ohlsen K , Morschhäuser J . 2013. Roles of different peptide transporters in nutrient acquisition in candida albicans. Eukaryot Cell 12:520–528. doi:10.1128/EC.00008-13 23376942 PMC3623439

[18] Lubkowitz MA , Hauser L , Breslav M , Naider F , Becker JM . 1997. An oligopeptide transport gene from candida albicans. Microbiology (Reading) 143 ( Pt 2):387–396. doi:10.1099/00221287-143-2-387 9043116

[19] Lubkowitz MA , Barnes D , Breslav M , Burchfield A , Naider F , Becker JM . 1998. Schizosaccharomyces pombe isp4 encodes a transporter representing a novel family of oligopeptide transporters. Mol Microbiol 28:729–741. doi:10.1046/j.1365-2958.1998.00827.x 9643541

[20] Bourbouloux A , Shahi P , Chakladar A , Delrot S , Bachhawat AK . 2000. Hgt1P, a high affinity glutathione transporter from the yeast Saccharomyces cerevisiae. J Biol Chem 275:13259–13265. doi:10.1074/jbc.275.18.13259 10788431

[21] Hauser M , Donhardt AM , Barnes D , Naider F , Becker JM . 2000. Enkephalins are transported by a novel eukaryotic peptide uptake system. J Biol Chem 275:3037–3041. doi:10.1074/jbc.275.5.3037 10652283

[22] 1982. The molecular and cellular biology of the yeast Saccharomyces: metabolism and gene expression. Cold Spring Harbor Laboratory Press, New York, NY.

[23] Cooper TG , Sumrada RA . 1983. What is the function of nitrogen catabolite repression in Saccharomyces cerevisiae? J Bacteriol 155:623–627. doi:10.1128/jb.155.2.623-627.1983 6135687 PMC217731

[24] Basrai MA , Zhang HL , Miller D , Naider F , Becker JM . 1992. Toxicity of oxalysine and oxalysine-containing peptides against candida albicans: regulation of peptide transport by amino acids. J Gen Microbiol 138:2353–2362. doi:10.1099/00221287-138-11-2353 1479355

[25] Cooper TG . 2002. Transmitting the signal of excess nitrogen in Saccharomyces cerevisiae from the Tor proteins to the GATA factors: connecting the dots. FEMS Microbiol Rev 26:223–238. doi:10.1111/j.1574-6976.2002.tb00612.x 12165425 PMC4384438

[26] Magasanik B , Kaiser CA . 2002. Nitrogen regulation in Saccharomyces cerevisiae. Gene 290:1–18. doi:10.1016/s0378-1119(02)00558-9 12062797

[27] Boer VM , Tai SL , Vuralhan Z , Arifin Y , Walsh MC , Piper MDW , de Winde JH , Pronk JT , Daran J-M . 2007. Transcriptional responses of Saccharomyces cerevisiae to preferred and nonpreferred nitrogen sources in glucose-limited chemostat cultures. FEMS Yeast Res 7:604–620. doi:10.1111/j.1567-1364.2007.00220.x 17419774

[28] Crépin L , Nidelet T , Sanchez I , Dequin S , Camarasa C . 2012. Sequential use of nitrogen compounds by Saccharomyces cerevisiae during wine fermentation: a model based on kinetic and regulation characteristics of nitrogen permeases. Appl Environ Microbiol 78:8102–8111. doi:10.1128/AEM.02294-12 22983966 PMC3485930

[29] Duc C , Pradal M , Sanchez I , Noble J , Tesnière C , Blondin B . 2017. A set of nutrient limitations trigger yeast cell death in a nitrogen-dependent manner during wine alcoholic fermentation. PLoS One 12:e0184838. doi:10.1371/journal.pone.0184838 28922393 PMC5602661

[30] Devia J , Bastías C , Kessi-Pérez EI , Villarroel CA , De Chiara M , Cubillos FA , Liti G , Martínez C , Salinas F . 2020. Transcriptional activity and protein levels of horizontally acquired genes in yeast reveal hallmarks of adaptation to fermentative environments. Front Genet 11:293. doi:10.3389/fgene.2020.00293 32425968 PMC7212421

[31] Bon EPS , Carvajal E , Stanbrough M , Rowen D , Magasanik B . 1997. Asparaginase II of Saccharomyces cerevisiae. Appl Biochem Biotechnol 63–65:203–212. doi:10.1007/BF02920425 9170245

[32] Island MD , Naider F , Becker JM . 1987. Regulation of dipeptide transport in Saccharomyces cerevisiae by micromolar amino acid concentrations. J Bacteriol 169:2132–2136. doi:10.1128/jb.169.5.2132-2136.1987 3553158 PMC212111

[33] Rai R , Genbauffe F , Lea HZ , Cooper TG . 1987. Transcriptional regulation of the DAl5 gene in Saccharomyces cerevisiae. J Bacteriol 169:3521–3524. doi:10.1128/jb.169.8.3521-3524.1987 3301804 PMC212427

[34] Wiles AM , Cai H , Naider F , Becker JM . 2006. Nutrient regulation of oligopeptide transport in Saccharomyces Cerevisiae. Microbiology 152:3133–3145. doi:10.1099/mic.0.29055-0 17005992

[35] Homann OR , Cai H , Becker JM , Lindquist SL . 2005. Harnessing natural diversity to probe metabolic pathways. PLoS Genet 1:e80. doi:10.1371/journal.pgen.0010080 16429164 PMC1342634

[36] Byrd C , Turner GC , Varshavsky A . 1998. The N-end rule pathway controls the import of peptides through degradation of a transcriptional repressor. EMBO J 17:269–277. doi:10.1093/emboj/17.1.269 9427760 PMC1170377

[37] Turner GC , Du F , Varshavsky A . 2000. Peptides accelerate their uptake by activating a ubiquitin-dependent proteolytic pathway. Nature 405:579–583. doi:10.1038/35014629 10850718

[38] Hauser M , Narita V , Donhardt AM , Naider F , Becker JM . 2001. Multiplicity and regulation of genes encoding peptide transporters in Saccharomyces cerevisiae. Mol Membr Biol 18:105–112. 11396605

[39] Du F , Navarro-Garcia F , Xia Z , Tasaki T , Varshavsky A . 2002. Pairs of dipeptides synergistically activate the binding of substrate by ubiquitin ligase through dissociation of its autoinhibitory domain. Proc Natl Acad Sci U S A 99:14110–14115. doi:10.1073/pnas.172527399 12391316 PMC137845

[40] Duteurtre B , Bourgeois C , Chollot B . 1971. Study of the assimilation of proline by brewing yeasts. J Inst Brew 77:28–35. doi:10.1002/j.2050-0416.1971.tb03350.x

[41] Thomas D , Surdin-Kerjan Y . 1997. Metabolism of sulfur amino acids in Saccharomyces cerevisiae. Microbiol Mol Biol Rev 61:503–532. doi:10.1128/mmbr.61.4.503-532.1997 9409150 PMC232622

[42] Salmon JM , Barre P . 1998. Improvement of nitrogen assimilation and fermentation kinetics under enological conditions by derepression of alternative nitrogen-assimilatory pathways in an industrial Saccharomyces cerevisiae strain. Appl Environ Microbiol 64:3831–3837. doi:10.1128/AEM.64.10.3831-3837.1998 9758807 PMC106562

[43] Fiechter G , Pavelescu D , Mayer HK . 2011. UPLC analysis of free amino acids in wines: profiling of on-lees aged wines. J Chromatogr B Analyt Technol Biomed Life Sci 879:1361–1366. doi:10.1016/j.jchromb.2011.02.005 21371950

[44] Sprouffske K , Wagner A . 2016. Growthcurver: an R package for obtaining interpretable metrics from microbial growth curves. BMC Bioinformatics 17:172. doi:10.1186/s12859-016-1016-7 27094401 PMC4837600

