# Supplementary material for: Unlocking the secrets of peptide transport in wine yeast: insights into oligopeptide transporter functions and nitrogen source preferences
Source: Appl Environ Microbiol. 2023 Oct 16;89(11):e01141-23. doi: 10.1128/aem.01141-23 (PMC10686055; doi:10.1128/aem.01141-23)
Supplement: Supplemental Tables and Figures — Tables S1-7 and Figures S1-7. [file aem.01141-23-s0001.docx]

# Supplemental Materials

**Tables S1-5 – Mean values of growth and fermentation parameters within the different media.** An analysis of variance followed by a Tukey's test (p-value = 0.05) was performed to indicate the significant differences among the values, which are represented in the "groups" column. Significant differences among the strains within the same condition are indicated with low case letters, and significant differences between conditions for the same strain are indicated with upper case letters. ‘sd’: standard deviation.

**Table S1 - Maximum rate of CO_2_ production (Vmax)**.

|  | **Vmax** | | | | | | | | |
| --- | --- | --- | --- | --- | --- | --- | --- | --- | --- |
|  | **NAP200** | | | **NA100** | | | **P200** | | |
|  | Vmax (g/L/h) | sd | groups | Vmax (g/L/h) | sd | groups | Vmax (g/L/h) | sd | groups |
| 59A (wt) | 0.969 | 0.109 | ab,A | 0.570 | 0.040 | a,B | 1.024 | 0.039 | a,A |
| Fot1 | 1.059 | 0.005 | a,A | 0.535 | 0.000 | a,B | 1.016 | 0.018 | a,A |
| Fot1Fot2 | 1.065 | 0.004 | a,A | 0.586 | 0.041 | a,B | 1.053 | 0.042 | a,A |
| fot1fot2Δ | 0.762 | 0.080 | abc,A | 0.545 | 0.013 | a,A | 0.703 | 0.079 | c,A |
| Fot2 | 0.997 | 0.038 | ab,A | 0.583 | 0.008 | a,B | 0.906 | 0.034 | ab,A |
| Fot3 | 1.119 | 0.074 | a,A | 0.569 | 0.008 | a,B | 0.969 | 0.027 | a,A |
| Opt1 | 0.561 | 0.217 | c,A | 0.642 | 0.017 | a,A | - | - | - |
| opt1Δ | 0.939 | 0.098 | abc,A | 0.542 | 0.039 | a,B | 1.071 | 0.090 | a,A |
| Opt2 | 0.803 | 0.145 | abc,A | 0.583 | 0.032 | a,A | 0.781 | 0.027 | bc,A |
| opt2Δ | 0.987 | 0.167 | ab,AB | 0.580 | 0.057 | a,B | 1.060 | 0.010 | a,A |
| PepKO | 0.601 | 0.033 | bc,A | 0.589 | 0.027 | a,A | - | - | - |

**Table S2 -** **Time to reach 80% of attenuation**.

|  | **Time to reach 80% of attenuation** | | | | | | | | |
| --- | --- | --- | --- | --- | --- | --- | --- | --- | --- |
|  | **NAP200** | | | **NA100** | | | **P200** | | |
|  | Time (h) | sd | groups | Time (h) | sd | groups | Time (h) | sd | groups |
| 59A (wt) | 150 | 12 | b,B | 258 | 30 | a,A | 144 | 0 | b,B |
| Fot1 | 120 | 0 | b,C | 264 | 0 | a,A | 144 | 0 | b,B |
| Fot1Fot2 | 144 | 0 | b,B | 216 | 0 | a,A | 144 | 0 | b,B |
| fot1fot2Δ | 168 | 0 | ab,B | 264 | 0 | a,A | 180 | 17 | a,B |
| Fot2 | 144 | 0 | b,B | 264 | 0 | a,A | 144 | 0 | b,B |
| Fot3 | 120 | 0 | b,B | 240 | 34 | a,A | 144 | 0 | b,B |
| Opt1 | 240 | 34 | a,A | 240 | 34 | a,A | - | - | - |
| opt1Δ | 144 | 34 | b,B | 264 | 0 | a,A | 132 | 17 | b,B |
| Opt2 | 168 | 34 | ab,A | 240 | 34 | a,A | 180 | 17 | a,A |
| opt2Δ | 144 | 34 | b,B | 264 | 0 | a,A | 144 | 0 | b,B |
| PepKO | 216 | 0 | a,A | 240 | 34 | a,A | - | - | - |

**Table S3 - Time to produce 1g of CO_2_**.

|  | **Time to produce 1 g of CO2** | | | | | | | | |
| --- | --- | --- | --- | --- | --- | --- | --- | --- | --- |
|  | **NAP200** | | | **NA100** | | | **P200** | | |
|  | Time (h) | sd | groups | Time (h) | sd | groups | Time (h) | sd | groups |
| 59A (wt) | 14 | 3 | a,B | 12 | 0 | a,B | 18 | 0 | b,A |
| Fot1 | 15 | 4 | a,A | 18 | 0 | a,A | 18 | 0 | b,A |
| Fot1Fot2 | 12 | 0 | a,A | 15 | 4 | a,A | 18 | 0 | b,A |
| fot1fot2Δ | 15 | 4 | a,A | 18 | 0 | a,A | 18 | 0 | b,A |
| Fot2 | 12 | 0 | a,A | 12 | 0 | a,A | 15 | 4 | b,A |
| Fot3 | 12 | 0 | a,A | 15 | 4 | a,A | 18 | 0 | b,A |
| Opt1 | 12 | 0 | a,A | 12 | 0 | a,A | 24 | 8 | ab,A |
| opt1Δ | 9 | 4 | a,A | 15 | 4 | a,A | 18 | 0 | b,A |
| Opt2 | 12 | 0 | a,A | 15 | 4 | a,A | 18 | 0 | b,A |
| opt2Δ | 15 | 4 | a,A | 15 | 4 | a,A | 18 | 0 | b,A |
| PepKO | 15 | 4 | a,AB | 12 | 0 | a,B | 36 | 8 | a,A |

**Table S4 - Maximum population (k).**

|  | **Maximum population (k)** | | | | | | | | |
| --- | --- | --- | --- | --- | --- | --- | --- | --- | --- |
|  | **NAP200** | | | **NA100** | | | **P200** | | |
|  | k | sd | groups | k | sd | groups | k | sd | groups |
| 59A (wt) | 10.186 | 0.563 | a,A | 7.716 | 0.335 | abc,B | 10.215 | 0.936 | abc,A |
| Fot1 | 9.812 | 0.543 | ab,A | 7.167 | 0.049 | bc,B | 9.457 | 0.241 | abc,A |
| Fot1Fot2 | 9.613 | 0.502 | ab,A | 7.279 | 0.076 | abc,B | 9.328 | 0.172 | bc,A |
| fot1fot2Δ | 8.357 | 0.544 | ab,AB | 7.219 | 0.046 | bc,B | 8.876 | 0.097 | c,A |
| Fot2 | 9.139 | 0.346 | ab,A | 7.167 | 0.182 | bc,B | 9.385 | 0.059 | abc,A |
| Fot3 | 9.980 | 0.172 | ab,A | 7.137 | 0.070 | c,C | 8.971 | 0.346 | c,B |
| Opt1 | 7.449 | 1.266 | b,A | 8.026 | 0.253 | abc,A | - | - | - |
| opt1Δ | 10.877 | 0.608 | a,A | 8.168 | 0.505 | a,B | 11.211 | 0.046 | a,A |
| Opt2 | 9.660 | 0.881 | ab,A | 8.078 | 0.182 | ab,A | 9.714 | 0.503 | abc,A |
| opt2Δ | 10.669 | 1.008 | a,A | 8.140 | 0.058 | a,B | 11.139 | 0.135 | ab,A |
| PepKO | 7.549 | 0.020 | b,A | 7.417 | 0.021 | abc,B | - | - | - |

**Table S5 - Maximum growth rate (r).**

|  | **Maximum growth rate (r)** | | | | | | | | |
| --- | --- | --- | --- | --- | --- | --- | --- | --- | --- |
|  | **NAP200** | | | **NA100** | | | **P200** | | |
|  | r | sd | groups | r | sd | groups | r | sd | groups |
| 59A (wt) | 0.110 | 0.025 | a,A | 0.107 | 0.023 | a,A | 0.103 | 0.038 | a,A |
| Fot1 | 0.112 | 0.006 | a,A | 0.096 | 0.007 | a,A | 0.105 | 0.005 | a,A |
| Fot1Fot2 | 0.113 | 0.012 | a,A | 0.100 | 0.017 | a,A | 0.113 | 0.005 | a,A |
| fot1fot2Δ | 0.087 | 0.008 | a,A | 0.088 | 0.014 | a,A | 0.057 | 0.006 | a,A |
| Fot2 | 0.111 | 0.011 | a,A | 0.094 | 0.019 | a,A | 0.096 | 0.001 | a,A |
| Fot3 | 0.128 | 0.016 | a,A | 0.099 | 0.013 | a,A | 0.107 | 0.010 | a,A |
| Opt1 | 0.113 | 0.020 | a,A | 0.119 | 0.020 | a,A | - | - | - |
| opt1Δ | 0.121 | 0.015 | a,A | 0.112 | 0.009 | a,A | 0.113 | 0.005 | a,A |
| Opt2 | 0.110 | 0.003 | a,A | 0.123 | 0.005 | a,A | 0.081 | 0.003 | a,B |
| opt2Δ | 0.117 | 0.020 | a,A | 0.111 | 0.019 | a,A | 0.115 | 0.009 | a,A |
| PepKO | 0.096 | 0.009 | a,A | 0.089 | 0.007 | a,A | - | - | - |

**Table S6 - Analysis of variance in gene expression.** ANOVA model: expression~Strain*Media*Time. Tukey's tests assign groups according to the significant differences found between the mean values by strain, media or timepoint.

| **By strain** | | | | | | | | | | |
| --- | --- | --- | --- | --- | --- | --- | --- | --- | --- | --- |
|  | ***FOT1*** | | ***FOT2*** | | ***OPT1*** | | ***OPT2*** | | ***Dal5*** | |
| Strain | Mean Value | Groups | Mean Value | Groups | Mean Value | Groups | Mean Value | Groups | Mean Value | Groups |
| 59A (wt) | 0.951 | b | 0.705 | a | 1.078 | b | 0.540 | b | 0.354 | b |
| Opt2Δ | 2.350 | a | 0.918 | a | 1.845 | a | - | - | 0.899 | a |
| Fot1Fot2Δ | - | - | - | - | 1.094 | b | 2.364 | a | 0.811 | a |
| Opt1 | - | - | - | - | 1.340 | b | - | - | - | - |
|  |  |  |  |  |  |  |  |  |  |  |
| **By media** | | | | | | | | | | |
|  | ***FOT1*** | | ***FOT2*** | | ***OPT1*** | | ***OPT2*** | | ***DAL5*** | |
| Medium | Mean Value | Groups | Mean Value | Groups | Mean Value | Groups | Mean Value | Groups | Mean Value | Groups |
| NAP200 | 2.245 | a | 0.342 | b | 0.646 | b | 0.884 | b | 0.370 | b |
| P200 | 1.966 | a | 1.206 | a | 0.827 | b | 2.581 | a | 0.960 | a |
| P200-SO4 | 0.741 | b | 0.886 | ab | 2.513 | a | 0.891 | b | 0.752 | a |
|  |  |  |  |  |  |  |  |  |  |  |
| **By timepoint** | | | | | | | | | | |
|  | ***FOT1*** | | ***FOT2*** | | ***OPT1*** | | ***OPT2*** | | ***DAL5*** | |
| Timepoint | Mean Value | Groups | Mean Value | Groups | Mean Value | Groups | Mean Value | Groups | Mean Value | Groups |
| 18 | 0.466 | b | 0.353 | b | 0.722 | b | 0.620 | b | 0.458 | b |
| 48 | 2.835 | a | 1.286 | a | 1.984 | a | 2.284 | a | 0.935 | a |

**Table S7 - Mass spectrometric data of all di-heptapeptides followed during this study.** RT: retention time; m/z: mass to charge ratio; CCS: collisional cross section; RSD: relative standard deviation. Peptide candidates with a unique sequence (i.e., no other possible peptide sequences with the same AA composition found in BSA) are underscored.

| Peptide candidate | RT (min) | Observed m/z | Charge | CCS (Å^2^) | Average Original Intensity (NAP200) | RSD | Average Original Intensity (P200) | RSD |
| --- | --- | --- | --- | --- | --- | --- | --- | --- |
| AI | 2.03 | 203.1389 | 1 | 154.86 | 684 | 8% | 1052 | 10% |
| DI | 0.71 | 247.1288 | 1 | 155.86 | 1635 | 7% | 2784 | 5% |
| FK | 1.49 | 294.1811 | 1 | 167.80 | 1718 | 7% | 3170 | 5% |
| FW | 4.92 | 352.1656 | 1 | 181.90 | 3346 | 10% | 6671 | 7% |
| FY | 3.72 | 329.1495 | 1 | 182.71 | 1141 | 6% | 1645 | 4% |
| IE | 1.13 | 261.1445 | 1 | 158.67 | 981 | 10% | 1431 | 7% |
| KT | 1.25 | 248.1604 | 1 | 155.81 | 1005 | 9% | 1628 | 7% |
| LL | 3.74 | 245.1860 | 1 | 166.49 | 8075 | 11% | 13052 | 9% |
| PL | 1.00 | 233.1495 | 1 | 158.32 | 988 | 11% | 1839 | 8% |
| PP | 2.72 | 229.1546 | 1 | 155.06 | 5885 | 5% | 9824 | 4% |
| RF | 0.83 | 213.1233 | 1 | 147.39 | 1492 | 6% | 2838 | 5% |
| SF | 0.83 | 338.1823 | 1 | 176.91 | 839 | 9% | 1500 | 6% |
| SI | 2.40 | 253.1183 | 1 | 159.04 | 654 | 7% | 1142 | 5% |
| TH | 1.88 | 219.1338 | 1 | 155.62 | 2503 | 13% | 4293 | 12% |
| TI | 1.25 | 257.1244 | 1 | 158.85 | 811 | 6% | 1346 | 5% |
| TM | 0.64 | 248.1604 | 1 | 155.81 | 979 | 5% | 1411 | 5% |
| VF | 1.10 | 251.1060 | 1 | 155.67 | 3623 | 9% | 6615 | 5% |
| VL | 3.37 | 265.1547 | 1 | 163.74 | 4160 | 7% | 6342 | 8% |
| VR | 2.83 | 231.1703 | 1 | 161.93 | 1482 | 13% | 2356 | 12% |
| AEF | 3.14 | 366.1661 | 1 | 183.27 | 5625 | 6% | 9727 | 5% |
| AFL | 4.59 | 350.2076 | 1 | 180.13 | 6334 | 7% | 11691 | 6% |
| DAF | 3.07 | 352.1503 | 1 | 178.25 | 683 | 12% | 1382 | 8% |
| DLL | 3.75 | 360.2129 | 1 | 192.76 | 808 | 6% | 1437 | 6% |
| FSA | 2.77 | 324.1554 | 1 | 175.60 | 3728 | 7% | 5820 | 6% |
| FSQ | 1.84 | 381.1769 | 1 | 188.33 | 1310 | 9% | 2372 | 6% |
| FTF | 5.21 | 414.2025 | 1 | 196.70 | 4211 | 9% | 8247 | 7% |
| FVE | 2.96 | 394.1973 | 1 | 191.66 | 3353 | 9% | 6158 | 8% |
| GEY | 1.87 | 368.1453 | 1 | 181.37 | 1633 | 5% | 2666 | 5% |
| IAE | 1.77 | 332.1816 | 1 | 175.31 | 2526 | 12% | 4690 | 8% |
| IAR | 0.79 | 359.2401 | 1 | 187.19 | 2544 | 8% | 4472 | 6% |
| IET | 2.09 | 362.1922 | 1 | 187.09 | 3294 | 8% | 5680 | 5% |
| IVR | 1.21 | 387.2714 | 1 | 197.52 | 1033 | 6% | 2004 | 8% |
| LFT | 3.92 | 380.2180 | 1 | 192.09 | 1671 | 7% | 2879 | 5% |
| LHT | 1.26 | 370.2085 | 1 | 196.19 | 2748 | 6% | 4894 | 6% |
| LIV | 4.55 | 344.2543 | 1 | 187.71 | 786 | 9% | 1620 | 8% |
| LLF | 5.92 | 392.2544 | 1 | 197.36 | 3883 | 9% | 8268 | 8% |
| LSQ | 1.07 | 347.1926 | 1 | 180.24 | 4443 | 7% | 7531 | 5% |
| LTA | 2.24 | 304.1866 | 1 | 170.97 | 540 | 10% | 1021 | 7% |
| LTE | 1.90 | 362.1923 | 1 | 183.40 | 8691 | 6% | 14156 | 5% |
| LVE | 2.45 | 360.2132 | 1 | 185.31 | 13478 | 5% | 21848 | 5% |
| LVN | 1.84 | 345.2136 | 1 | 182.14 | 831 | 10% | 1535 | 7% |
| LYE | 2.78 | 424.2081 | 1 | 198.31 | 9323 | 5% | 13865 | 4% |
| LYY | 4.12 | 458.2289 | 1 | 206.93 | 7833 | 7% | 13458 | 6% |
| TFI | 5.04 | 380.2180 | 1 | 193.97 | 743 | 10% | 1653 | 9% |
| TIS | 1.19 | 320.1816 | 1 | 173.95 | 1201 | 5% | 1867 | 11% |
| VAF | 3.97 | 336.1919 | 1 | 175.17 | 11297 | 6% | 18776 | 4% |
| VTD | 0.81 | 334.1609 | 1 | 173.44 | 5024 | 4% | 7147 | 4% |
| VVS | 1.09 | 304.1865 | 1 | 170.97 | 1690 | 6% | 2420 | 6% |
| ALVE | 3.21 | 431.2501 | 1 | 201.91 | 2512 | 8% | 4434 | 6% |
| FLGS | 3.65 | 423.2238 | 1 | 200.23 | 588 | 12% | 1201 | 8% |
| FVAF | 5.53 | 483.2603 | 1 | 212.09 | 913 | 11% | 1931 | 9% |
| IETM | 3.18 | 493.2330 | 1 | 215.74 | 5788 | 7% | 9550 | 6% |
| LGSF | 4.08 | 423.2241 | 1 | 198.33 | 7620 | 10% | 14327 | 6% |
| LIAF | 5.74 | 463.2916 | 1 | 212.61 | 663 | 10% | 1402 | 9% |
| LLYY | 5.12 | 571.3122 | 1 | 233.82 | 775 | 10% | 1724 | 9% |
| LTAD | 1.89 | 419.2137 | 1 | 194.67 | 1765 | 8% | 2861 | 6% |
| LTEF | 4.27 | 509.2609 | 1 | 217.31 | 2100 | 8% | 3800 | 6% |
| LVEL | 4.87 | 473.2973 | 1 | 216.26 | 8030 | 7% | 13870 | 6% |
| LVLI | 6.17 | 457.3385 | 1 | 218.66 | 1830 | 10% | 3780 | 9% |
| LVNE | 2.32 | 474.2560 | 1 | 210.37 | 3492 | 6% | 5374 | 5% |
| LVTD | 2.48 | 447.2451 | 1 | 203.38 | 4508 | 6% | 8247 | 6% |
| LVVS | 3.31 | 417.2708 | 1 | 200.41 | 2257 | 10% | 4266 | 7% |
| LYEI | 4.95 | 537.2921 | 1 | 230.57 | 849 | 10% | 1825 | 9% |
| VEVS | 2.17 | 433.2293 | 1 | 199.95 | 637 | 10% | 1170 | 8% |
| VVST | 1.80 | 405.2345 | 1 | 193.20 | 4559 | 9% | 8070 | 6% |
| LILNR | 4.03 | 314.7101 | 1 | 149.70 | 541 | 6% | 1020 | 8% |
| VFDKL | 4.18 | 311.1835 | 2 | 296.71 | 619 | 13% | 1251 | 12% |
| LLPKIE | 4.47 | 356.7332 | 2 | 318.21 | 831 | 11% | 1666 | 8% |
| TRKVPQ | 1.55 | 364.7238 | 2 | 314.44 | 3214 | 19% | 6547 | 9% |
| LLPKIET | 4.57 | 407.2571 | 2 | 312.72 | 721 | 14% | 1459 | 11% |
| LPKIETM | 4.59 | 416.2353 | 2 | 312.38 | 1181 | 12% | 2450 | 10% |
| LVEVSRS | 2.88 | 395.2263 | 2 | 306.43 | 891 | 10% | 1568 | 8% |


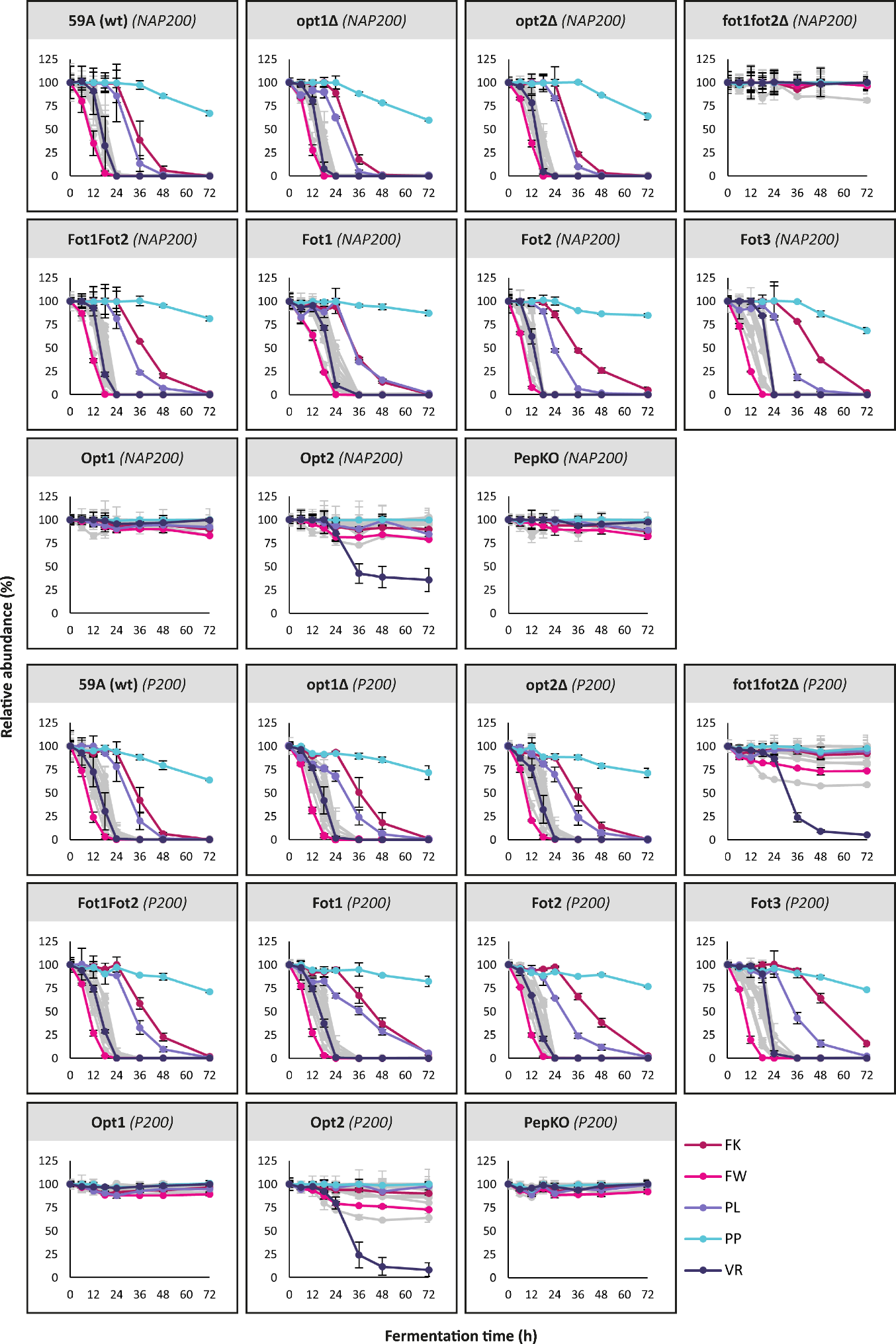


**Figure S1 - Dipeptide consumption curves of the strains tested on NAP200 and P200.** Five dipeptides are depicted in color to represent their peptide length group. All other dipeptide consumption curves are depicted as ‘shadow’ in the background.


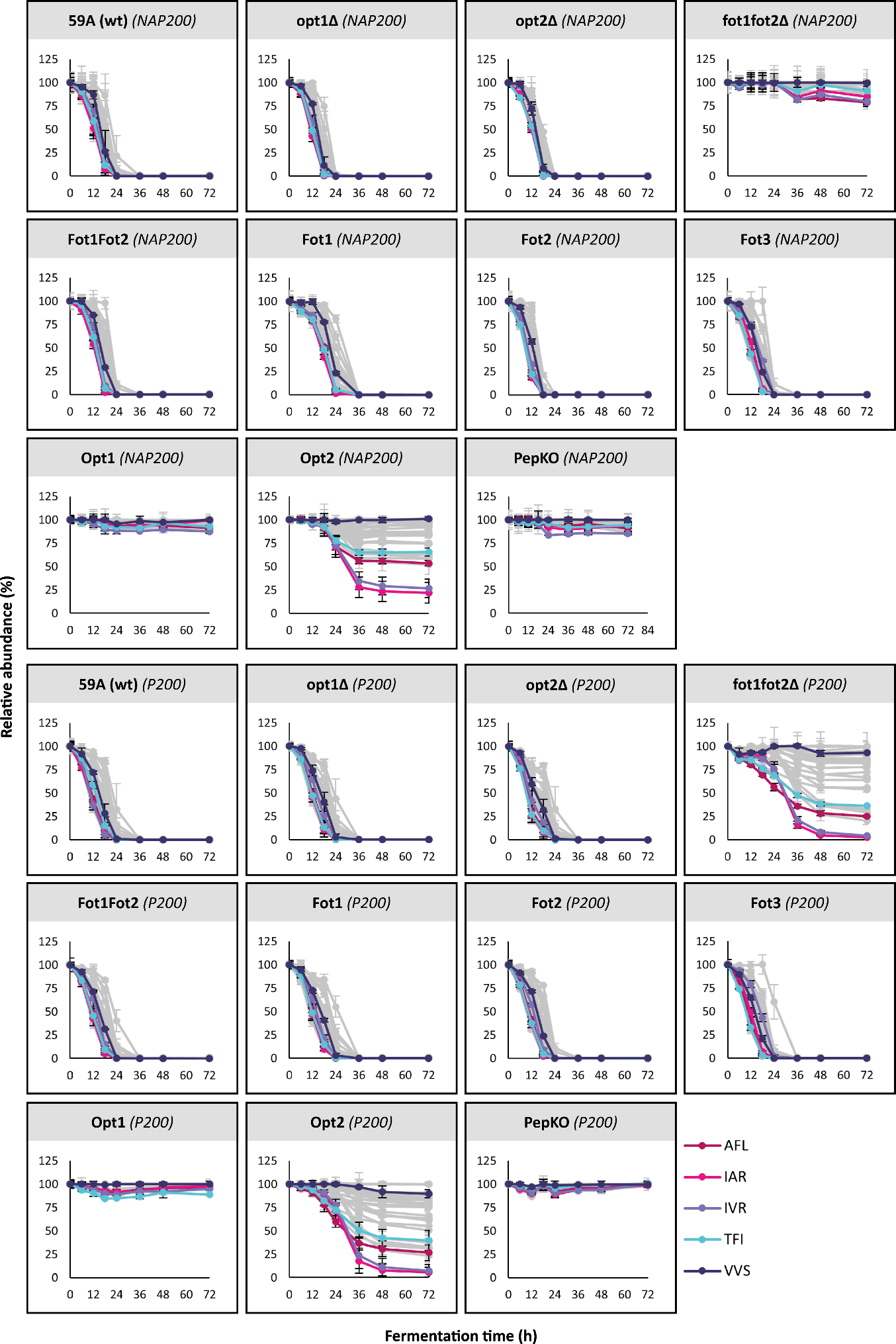


**Figure S2 -** **Tripeptide consumption curves of the strains tested on NAP200 and P200.** Five tripeptides are depicted in color to represent their peptide length group. All other tripeptide consumption curves are depicted as ‘shadow’ in the background.


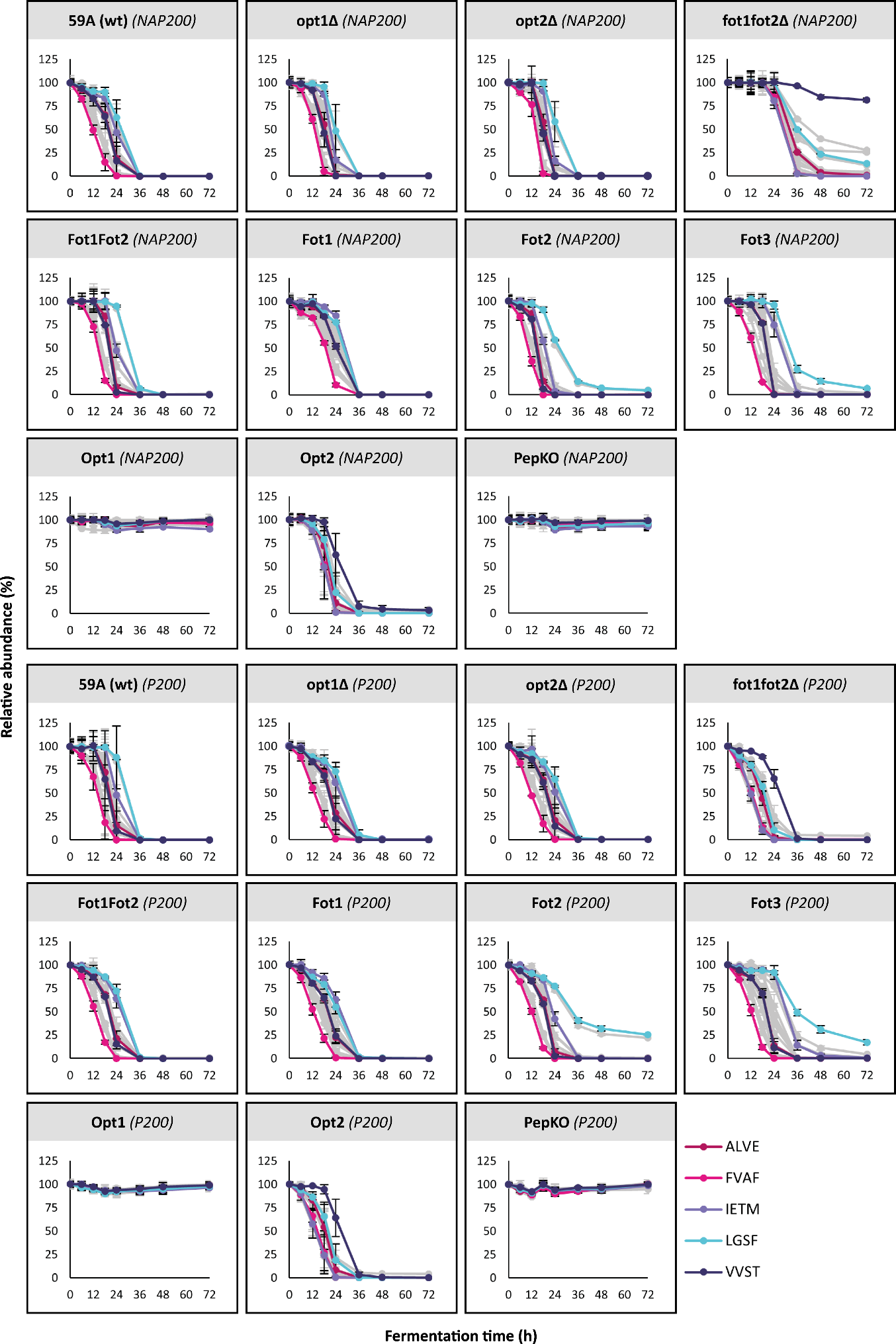


**Figure S3 - Tetrapeptide** **consumption curves of the strains tested on NAP200 and P200.** Five tetrapeptides are depicted in color to represent their peptide length group. All other tetrapeptide consumption curves are depicted as ‘shadow’ in the background.


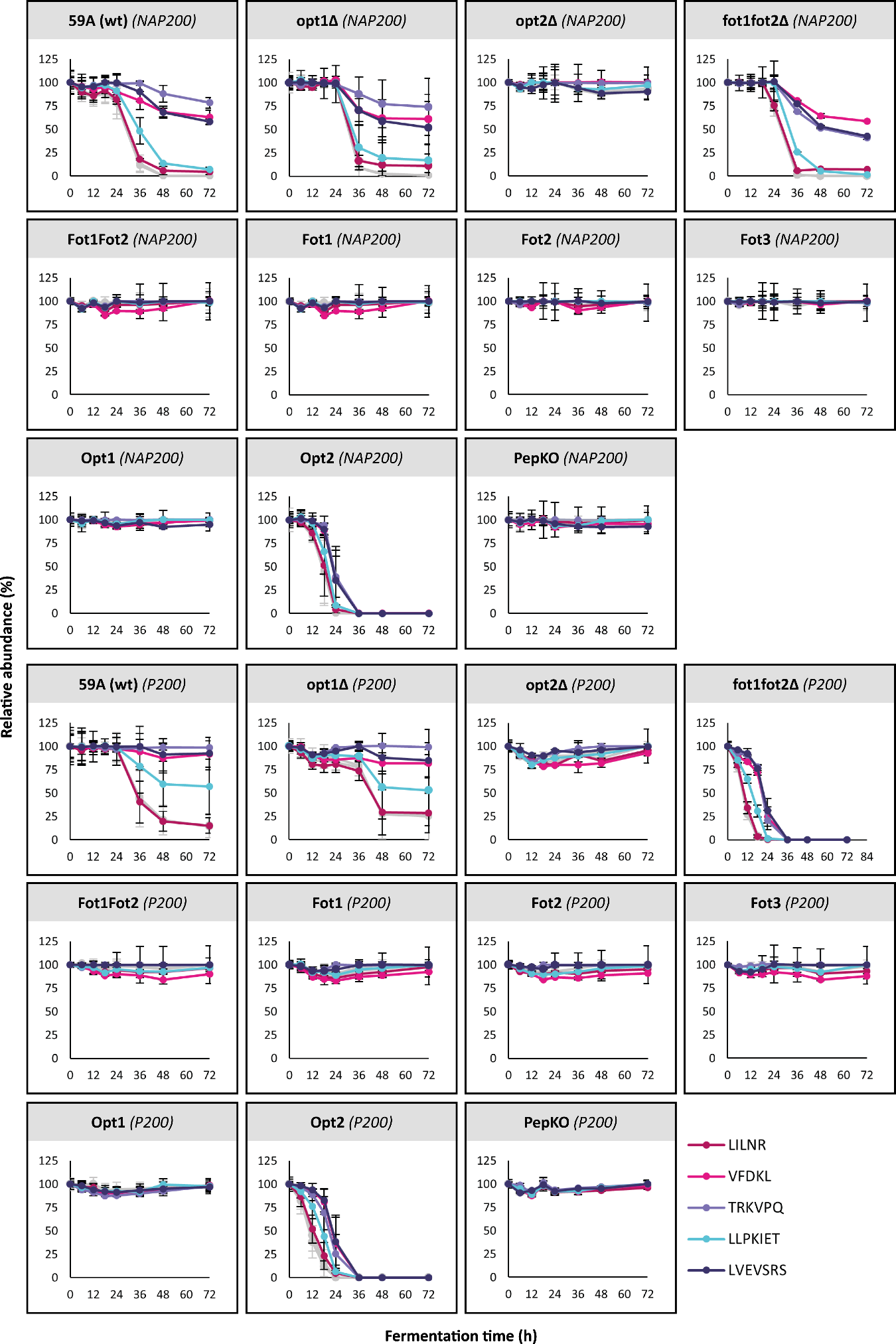


**Figure S4 - Penta-heptapeptide consumption curves of the strains tested on NAP200 and P200.** Five peptides are depicted in color to represent this peptide length group. All other penta- to heptapeptide consumption curves are depicted as ‘shadow’ in the background.


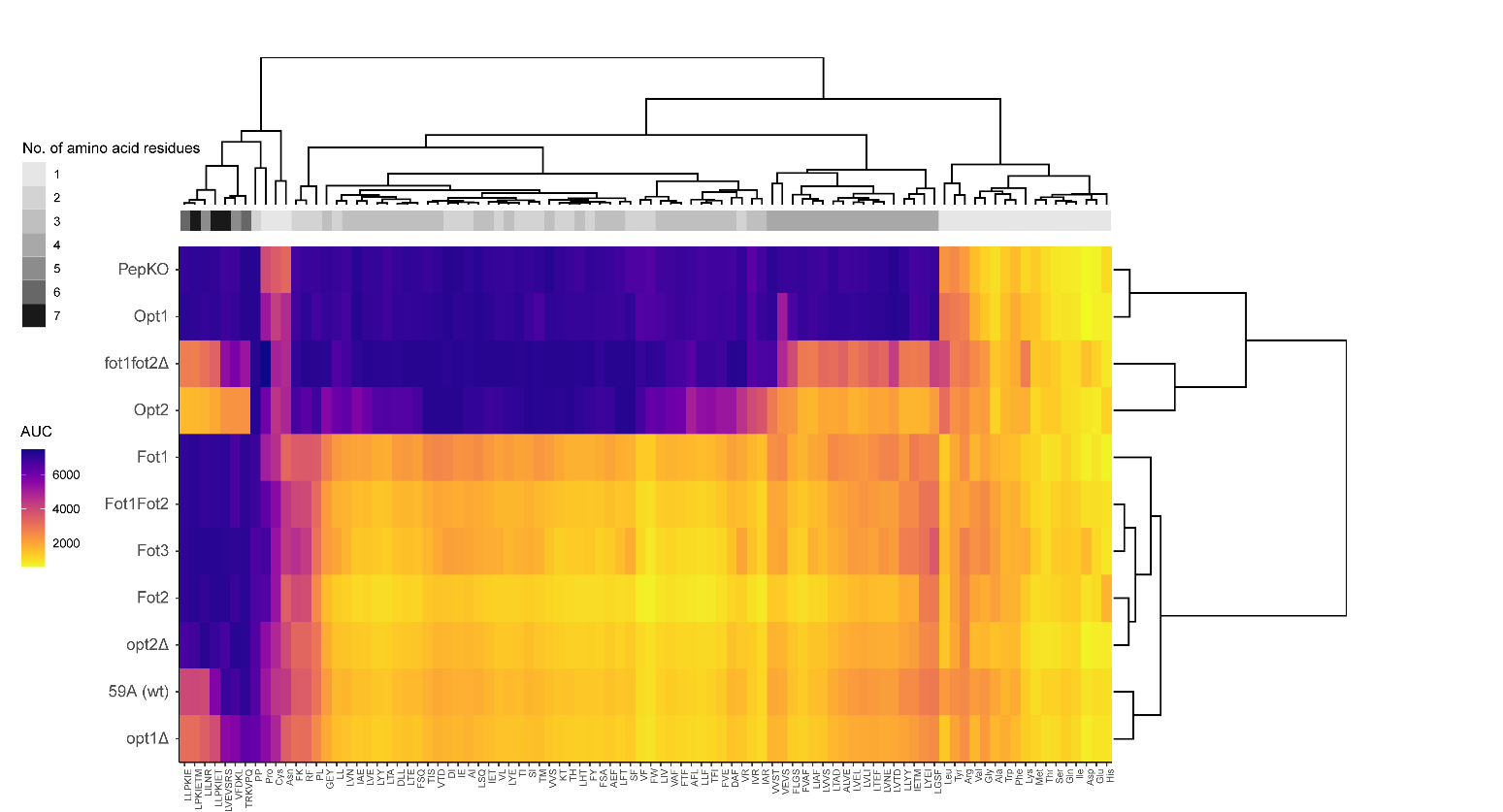


**Figure S5 - Consumption of FAA and peptides in NAP200.** The heatmap represents the consumption of FAA and di- to heptapeptides (columns) by the researched strains (rows). Strains are sorted by consumption preferences similarity, represented by the cladogram to the right. The cladogram on top of the heatmap ranks the FAA and di- to heptapeptides according to their preferential consumption.

***
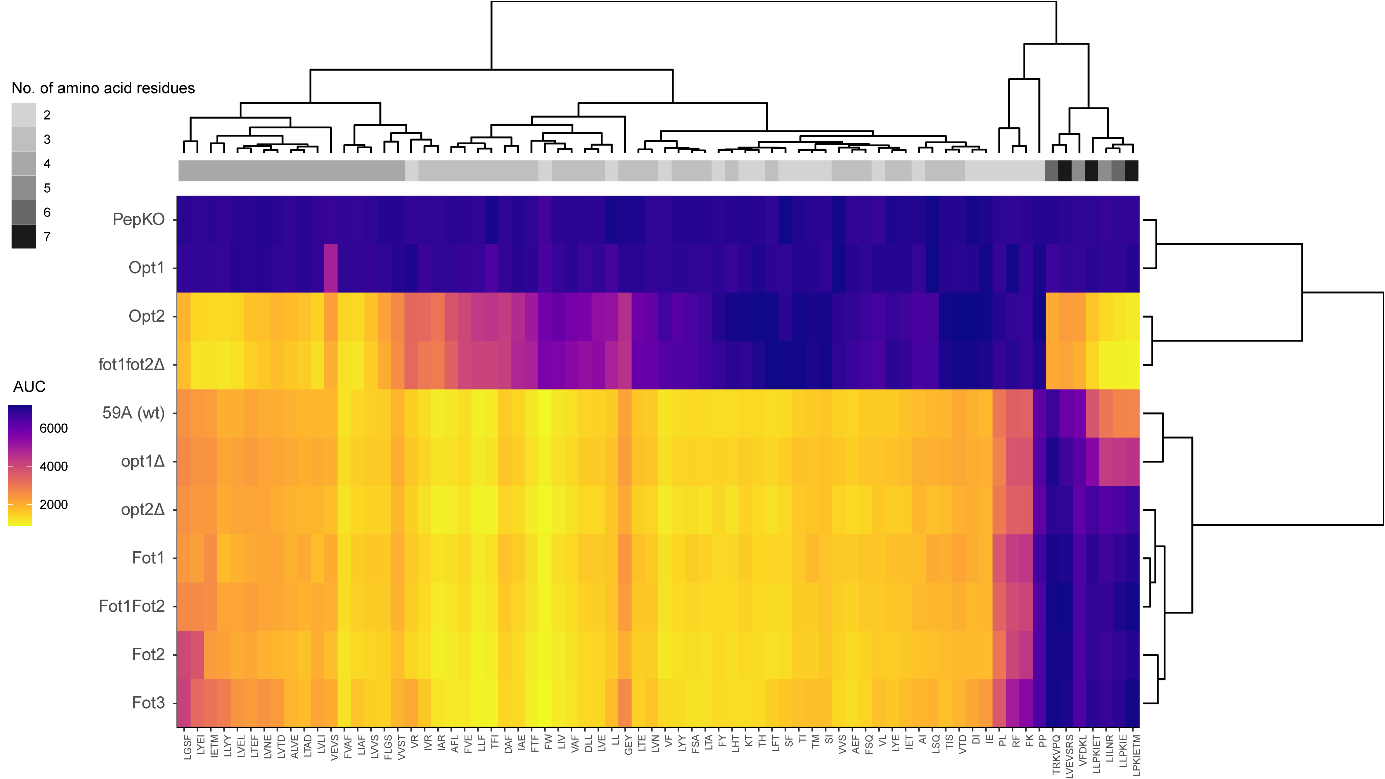
***

**Figure S6 - Consumption of peptides in P200.** The heatmap represents the consumption of di- to heptapeptides (columns) by the researched strains (rows). Strains are sorted by consumption preferences similarity, represented by the cladogram to the right. The cladogram on top of the heatmap ranks the di- to heptapeptides according to their preferential consumption.


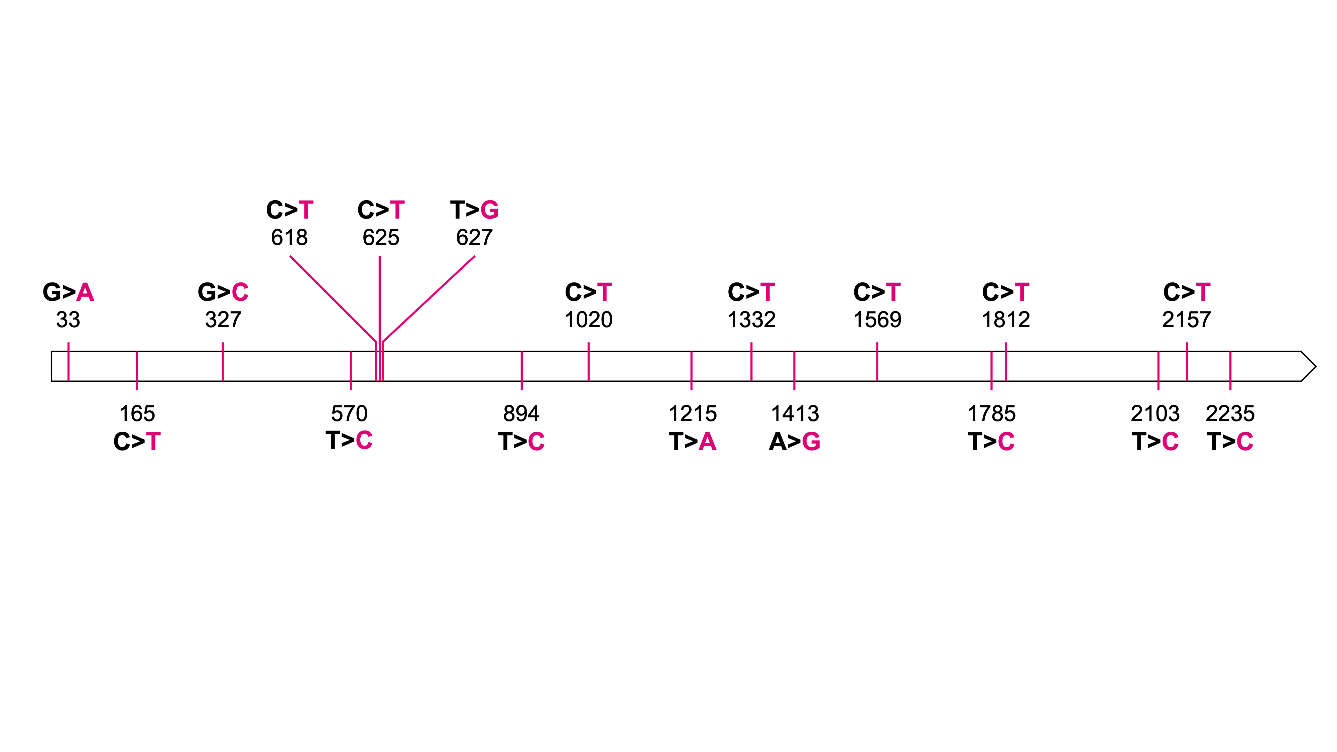


**Figure S7. Variable sites in OPT1 gene between S288c (in black) and 59A (in pink) strains.** Pairwise alignment between the two gene versions reports an identity percentage of 99.25%, with all these variable sites constituting synonymous mutations as the translated protein sequences are 100% identical.
